# LPS- or *Pseudomonas aeruginosa*-mediated activation of the macrophage TLR4 signaling cascade depends on membrane lipid composition

**DOI:** 10.7717/peerj.1663

**Published:** 2016-02-04

**Authors:** Axel Schoeniger, Herbert Fuhrmann, Julia Schumann

**Affiliations:** 1 Faculty of Veterinary Medicine, Institute of Physiological Chemistry, University of Leipzig, Leipzig, Germany; 2 Clinic for Anesthesiology and Surgical Intensive Care, University Hospital Halle (Saale), Halle (Saale), Germany

**Keywords:** CD14, TLR4, PUFA, Macrophages, Lipid rafts

## Abstract

It is well known that PUFA impede the LPS-mediated activation of the transcription factor NFkappaB. However, the underlying mode of action has not been clarified yet. To address this issue in a comprehensive approach, we used the monocyte/macrophage cell line RAW264.7 to investigate the consequences of a PUFA supplementation on the TLR4 pathway with a focus on (i) the gene expression of TLR4 itself as well as of its downstream mediators, (ii) the membrane microdomain localization of TLR4 and CD14, (iii) the stimulation-induced interaction of TLR4 and CD14. Our data indicate that the impairment of the TLR4-mediated cell activation by PUFA supplementation is not due to changes in gene expression of mediator proteins of the signaling cascade. Rather, our data provide evidence that the PUFA enrichment of macrophages affects the TLR4 pathway at the membrane level. PUFA incorporation into membrane lipids induces a reordering of membrane microdomains thereby affecting cellular signal transduction. It is important to note that this remodeling of macrophage rafts has no adverse effect on cell viability. Hence, microdomain disruption via macrophage PUFA supplementation has a potential as non-toxic strategy to attenuate inflammatory signaling.

## Introduction

The binding of ligands, e.g., lipopolysaccharide (LPS) or the gram-negative bacterium *Pseudomonas aeruginosa*, to the macrophage Toll-like receptor 4 (TLR4) activates the TLR4 signaling cascade, which eventually results in the activation of the nuclear factor kappa B (NF*κ*B) (McIsaac, Stadnyk & Lin, 2012). The main steps of the TLR4 pathway include the interaction of TLR4 with its co-receptor CD14, the activation of the adaptor protein MyD88, the recruitment of the IL-1 receptor associated kinase 4 (IRAK-4) to the TLR receptor complex, the phosphorylation-dependent activation of IRAK-1, the association of IRAK-1 with the TNF receptor-associated factor 6 (TRAF6) and subsequently the activation of the I kappa B kinase complex (IKK complex) which leads to the activation of NF*κ*B (McIsaac, Stadnyk & Lin, 2012). As a result, the macrophage polarizes into the inflammation-driving M1 phenotype (Sica & Mantovani, 2012). M1 macrophages are characterized by the synthesis and the release of pro-inflammatory cytokines, such as IL-1*β*, IL-6 or TNF-*α*, and are able to develop a severe respiratory burst thus contributing to the defense against microbial pathogens (Sica & Mantovani, 2012). However, an excessive inflammatory response by M1 macrophages, which is a hallmark of chronic infections with persistent pathogens, such as *P. aeruginosa*, is also reported to cause tissue damage, to lead to functional restrictions and to favor secondary infections (Park et al., 2007; Ambrozova, Pekarova & Lojek, 2010).

More recently a further functional phenotype of macrophages has been described: the M2 macrophage (Sica & Mantovani, 2012). M2 macrophages are characterized by a high phagocytic activity and the synthesis of anti-inflammatory cytokines (Sica & Mantovani, 2012). Macrophage polarization into the M2 type has been shown to be negatively associated with the microbial activity of the immune cells (Sica & Mantovani, 2012). M2 macrophages are involved in tissue remodeling and immune regulation (Sica & Mantovani, 2012). In addition, due to their anti-inflammatory properties M2 macrophages are believed to prevent detrimental M1 immune responses (Sica & Mantovani, 2012).

With the introduction of the lipid raft hypothesis the impact of the cellular membrane lipid composition has moved into the focus of biomedical science. It is assumed that membrane microdomains play a pivotal role in the initiation of signal transduction processes (Ye et al., 2010; Zhang, Zhang & Sun, 2010). In previous investigations, we already showed the membrane lipid composition to depend on the availability of fatty acids (Schumann et al., 2011; Basiouni et al., 2012). Supplementation of macrophages (cell line RAW264.7) with polyunsaturated fatty acids (PUFA) for 72 h in a concentration of 15 µM resulted in an incorporation of these fatty acids into both non-raft and lipid raft membrane domains (Schumann et al., 2011). This PUFA integration altered the physical-chemical properties of the membrane microdomains with the unsaturation index being positively related to the number of double bonds of a supplemented fatty acid (Schumann et al., 2011). Interestingly, a PUFA supplementation of cells has been shown by us and others to lead to an impairment of the LPS-mediated stimulation of NF*κ*B activity (Zhao et al., 2005; Ren & Chung, 2007; Weldon et al., 2007; Chang et al., 2010; Mullen, Loscher & Roche, 2010; Schumann & Fuhrmann, 2010). Accordingly, in a series of studies we previously demonstrated that enrichment of macrophages with PUFA of both the n-3 and the n-6 family (i) promotes the phagocytosis rate as well as the bactericidal capacity of the immune cells (Adolph, Fuhrmann & Schumann, 2012), (ii) diminishes the activation-induced respiratory burst (Adolph et al., 2012), and (iii) down-regulates the synthesis of the pro-inflammatory cytokines IL-1*β*, IL-6 and TNF-*α* (Schoeniger et al., 2011). A summarized presentation of the PUFA effects on macrophage cytokine synthesis can be found in Fig. S1. All together, the data underline that the enrichment of macrophages with PUFA leads to a polarization of the immune cells from the M1 type to the M2 type.

In the present study we aimed to elucidate the mechanisms underlying the PUFA effects. We were able to show that the impairment of the TLR4-mediated cell activation due to PUFA supplementation is not based on a modulation of the gene expression of mediator proteins of the signaling cascade. Rather, our data provide evidence that the PUFA enrichment of macrophages affects the TLR4 pathway at the membrane level.

## Materials and Methods

### Materials

All chemicals and reagents were obtained from Sigma-Aldrich (Taufkirchen, Germany) unless noted otherwise. Cell culture flasks were purchased from Greiner Bio-One (Frickenhausen, Germany). HEPES (25 mmol/L)-buffered RPMI 1640 culture medium containing 300 mg/L L-glutamine was acquired from Pan-Biotech (Aidenbach, Germany).

### Cell culture, fatty acid supplementation and stimulation

The mouse monocyte/macrophage cell line RAW264.7 (ATCC number TIB-71) was used. RAW264.7 were cultured in RPMI 1640 medium containing 4.5 g/L glucose, 5% v/v FCS and 0.2% v/v ethanol (basic medium). The fatty acids alpha-linolenic acid (LNA, C18:3n3), eicosapentaenoic acid (EPA, C20:5n3), docosahexaenoic acid (DHA, C22:6n3), linoleic acid (LA, C18:2n6) or arachidonic acid (AA, C20:4n6) (all Biotrend, Köln, Germany) were included in the culture medium in concentrations of 15 µmol/L using ethanol as a vehicle (0.2% v/v final ethanol concentration). Cells were cultured in the enriched media in 75 cm^2^ cell culture flasks totaling 72 h at 37°C and 5% CO_2_ in a humidified atmosphere. Stimulation of cells was performed in the last 24 h of fatty acid supplementation by addition of LPS (1 µg/mL, from *E. coli* serotype 0111:B4) or viable *P. aeruginosa* (ATCC 10145, growth restriction via gentamicin (10 µg/mL), bacterium/cell ratio 1:1). Periods of supplementation and stimulation were chosen in accordance with our previous investigations and were proven to result in a membrane fatty acid steady state as well as reproducible effects on macrophage functionality.

### Quantitative real-time PCR

RAW264.7 were cultured, supplemented and stimulated with LPS or *P. aeruginosa* ATCC 10145 as described above. Gene expression was analyzed by a SYBR Green-based quantitative real-time PCR. Total RNA was extracted using the RNeasy kit (Qiagen GmbH, Hilden, Germany) with an on-column DNase digestion. 1 µg RNA was reverse transcribed to cDNA using Oligo(dT)_12−18_Primers and SuperScript^®^ III Reverse Transcriptase (Invitrogen GmbH, Darmstadt, Germany), and quantitative real-time PCR was performed by means of suitable murine RT^2^ qPCR primer assays (SABiosciences, Hilden, Germany) and the SensiMix™ SYBR Kit (Bioline, Luckenwalde, Germany). Genes of interest amplified are TLR4, CD14, MyD88, IRAK-4 and TRAF6; housekeeping gene amplified is CASC3. Positive controls (XpressRef™ Universal Total RNA, SABiosciences, Frederick, USA) as well as negative controls (i.e., no template control) were performed in each run. Thermal cycling was carried out on the Rotor-Gene 6000 real-time PCR system (Qiagen GmbH, Hilden, Germany) at 95°C for 10 min followed by 45 cycles of 95°C for 15 s, 55°C for 30 s and 72°C for 15 s. Relative quantification was performed with the Rotor-Gene 6000 Series Software 1.7. The quantitative real-time PCR was performed in triplicate of three independent RNA isolations.

### Fluorescence microscopy

RAW264.7 were cultured on sterile coverslips in 12-well plates, supplemented and stimulated with LPS or *P. aeruginosa* as described above. The cells were fixed in 4% paraformaldehyde for 10 min, rinsed in PBS two times and permeabilized in 0.1% Triton-X100 in PBS for 5 min. Non-specific binding sites were blocked by incubation in 1% BSA in PBS for 1 h. Afterwards cells were incubated in a humidified chamber for 1 h at room temperature with species-specific primary antibodies against GM1, TLR4 (both Biozol, Eching, Germany) as well as CD14 (Santa Cruz Biotech., Heidelberg, Germany), rinsed in PBS three times and incubated for 1 h in a humidified chamber at room temperature in the dark with appropriate secondary antibodies conjugated to Alexa Fluor 350, Alexa Fluor 488 and Alexa Fluor 647 respectively (Invitrogen GmbH, Darmstadt, Germany). The coverslips with the labeled cells were rinsed in PBS three times, mounted onto glass slides and examined under a BZ-9000 fluorescence microscope (Keyence, Neu-Isenburg, Germany). Microscopic images were analyzed using the public domain software ImageJ (Version 1.47n). Co-localization was quantified using Manders’ coefficient as previously described (Manders, Verbeek & Aten, 1993). Four independent experiments were performed in triplicates for each combination of PUFA supplementation and stimulation of cells.

### Co-immunoprecipitation and Western blot

RAW264.7 were cultured, supplemented and stimulated with LPS or *P. aeruginosa* as described above. The cells were lysed in ice-cold lysis buffer (50 mM Hepes, 100 mM NaCl, 2 mM EDTA, 10% glycerol, 1% Igepal CA-630) in presence of the protease inhibitor cocktail Mammalian Protease Arrest (VWR, Darmstadt, Germany) in a final concentration of 0.2% v/v. Cell lysates with a total protein concentration of 100 µg/mL were incubated with 1 µg of an anti-mouse CD14 antibody (Abnova, Heidelberg, Germany) at 4°C for 1 h. Immune complexes were captured with 20 µl Protein A/G Plus–Agarose beads (Santa Cruz Biotech., Heidelberg, Germany) at 4°C overnight and under constant rotation. Agarose beads were washed 3 times with lysis buffer, resuspended in 35 µl Roti-Load 1 sample buffer (Roth, Karlsruhe, Germany) and boiled for 10 min. Immunoprecipitated proteins were separated by SDS-PAGE using the Amersham ECL Gel box and the Amersham ECL Gel 4–12% (both VWR, Darmstadt, Germany), and subsequently blotted on a 0.22 µm nitrocellulose membrane (Advansta, Menlo Park, USA) for Western blot analysis. The membrane was blocked in PBS containing 0.05% Tween20 and 5% nonfat dry milk and divided into two parts (upper part ranged from 170 kDa to 70 kDa, lower part ranged from 70 kDa to 10 kDa). Immunostaining was performed overnight at 4°C using appropriated species-specific primary antibodies against TLR4 (=upper part, Invitrogen GmbH, Darmstadt, Germany) and CD14 (=lower part, Santa Cruz Biotech., Heidelberg, Germany), followed by a 2 h incubation at room temperature with appropriate horseradish peroxidase conjugated secondary antibodies (Southern Biotech, Berlin, Germany and Dianova, Hamburg, Germany respectively). Bands were visualized using the Clarity ECL western blotting substrate (Bio-Rad, München, Germany) according to the manufacturer’s instructions. Band specificity was verified by performing IgG controls as well as by checking protein size using the PageRuler prestained protein ladder (Thermo Fisher Scientific Inc., Waltham, MA, USA). Band intensities were determined densitometrically using the GeneTools image analysis software from Syngene (Version 4.01, Cambridge, United Kingdom). Four independent experiments were performed in duplicate for each combination of PUFA supplementation and stimulation of cells.

### Statistical analysis

Data are shown as mean ± standard deviation (S.D.). Two-way analysis of variance followed by unpaired Students *t* test was used to identify significant differences between means. The statistical analysis was carried out by means of the program GraphPad Prism 6 (GaphPad Software, La Jolla, CA, USA). In all cases, *p* < 0.05 was considered to indicate significant differences.

## Results and Discussion

It is well known that PUFA impede the LPS-mediated activation of the transcription factor NF*κ*B, however, the underlying mechanisms have not been elucidated yet. Nevertheless, according to scientific literature there are two possible scenarios. On the one hand unsaturated fatty acids are discussed to diminish the gene expression of the TLR4 receptor (Lu et al., 2007; Liu et al., 2012). On the other hand PUFA are reported to disrupt membrane microdomain composition (Parker et al., 2008; Wong et al., 2009; Shaikh, Jolly & Chapkin, 2012), which might hamper the stimulation-induced initiation of the TLR4 signaling cascade via blocking the TLR4-CD14 interaction in lipid rafts. To address this issue in a comprehensive approach we investigated the consequences of a PUFA supplementation on the TLR4 pathway with a focus on (i) the gene expression of TLR4 itself as well as of its downstream mediators, (ii) the membrane microdomain localization of TLR4 and CD14, (iii) the stimulation-induced interaction of TLR4 and CD14 using the monocyte/macrophage cell line RAW264.7. For the first time the effects of various fatty acids differing in their family (n-3 versus n-6), chain length and number of double bonds, were analyzed in parallel. Of note, PUFA concentration used for supplementation (15 µM) matches physiological conditions (Corsetto et al., 2012). Biological relevance of gained results was further improved by the use of the viable gram-negative pathogen *P. aeruginosa* ATCC 10145 as stimulus. Supplementation and stimulation protocols were in accordance with previous investigations of the working group. A supplementation period of 72 h is needed to complete the incorporation of the fatty acids into the membrane of RAW264.7 cells. A stimulation period of 24 h is proven to result in macrophage (M1) activation, which is characterized by an increase in reactive oxygen intermediates (Adolph et al., 2012) as well as pro-inflammatory cytokines (Fig. S1) (Schoeniger et al., 2011).

### PUFA have no effect on gene expression of downstream mediator proteins of the TLR4 pathway

The impact of stimulation and/or PUFA supplementation on the expression of key mediators of the TLR4 pathway was analyzed by quantitative real-time PCR.

**Figure 1:**
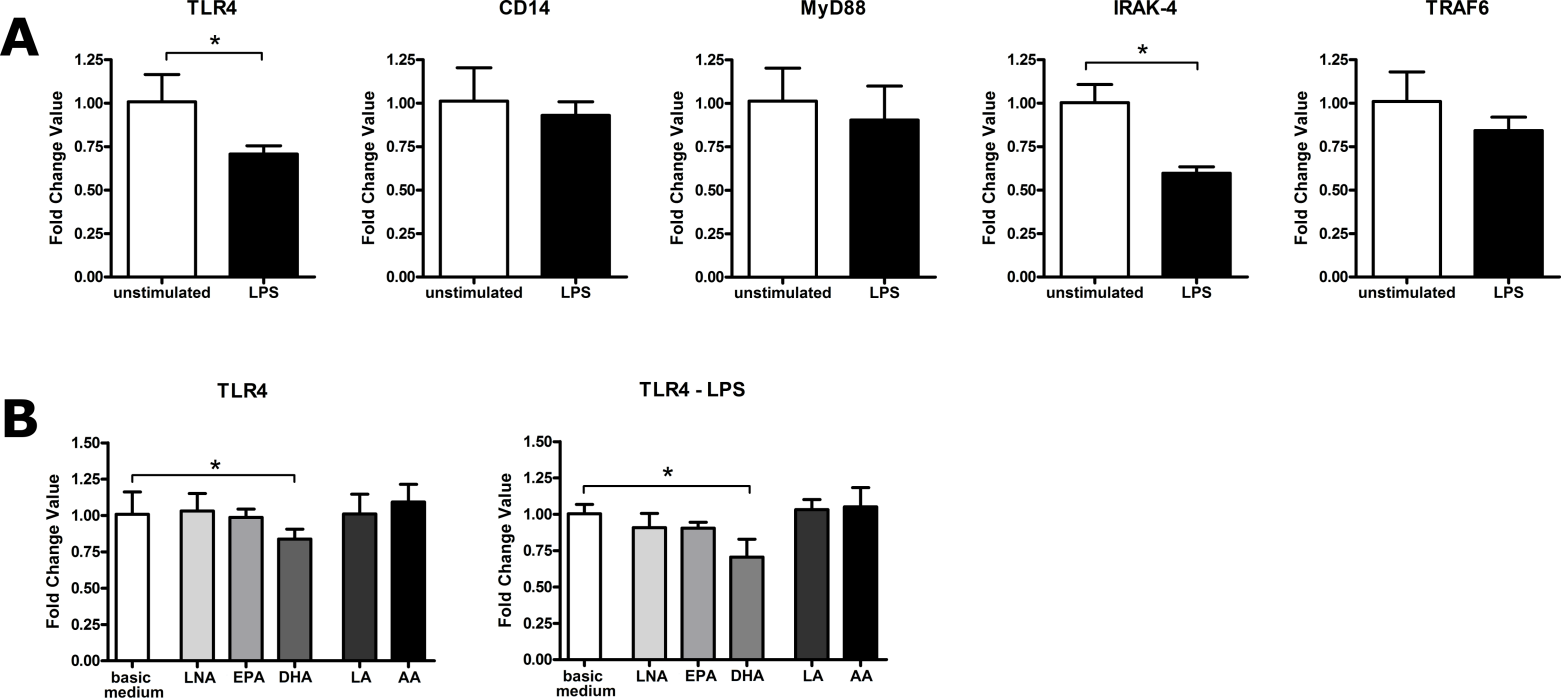
LPS- or PUFA-mediated modulation of gene expression of TLR4, CD14, MyD88, IRAK-4 and TRAF6 in RAW264.7 macrophages. Gene expression was determined by quantitative real-time PCR of total RNA isolated from RAW264.7 macrophages using the housekeeping gene CASC3 for normalization of mRNA expression levels. Data are expressed as mean ± S.D. (*N* = 3, *n* = 3). Asterisks indicate a statistically significant difference compared with the unstimulated or unsupplemented controls (^∗^*p* < 0.05, ^∗∗^*p* < 0.01). (A) Cells were cultured in basic medium (RPMI 1640 containing 4.5 g/L glucose, 5% v/v FCS, 0.2% v/v ethanol) and stimulated with LPS (1 µg/mL) for 24 h. (B) Cells were cultured in basic medium supplemented with alpha-linolenic acid (LNA), eicosapentaenoic acid (EPA), docosahexaenoic acid (DHA), linoleic acid (LA) or arachidonic acid (AA) in a concentration of 15 µM for 72 h. Cell stimulation was performed by addition of LPS (1 µg/mL) to the culture medium in the last 24 h of incubation.

Stimulation of RAW264.7 cultured in basic medium with LPS or viable *P. aeruginosa* resulted in a moderate but significant decrease in the expression of the TLR4 gene (Fig. 1A). Likewise, IRAK-4 gene expression was down-regulated upon RAW264.7 stimulation (Fig. 1A). There were no changes in the gene expression level of CD14, MyD88 or TRAF6 following treatment of the macrophages with LPS and viable *P. aeruginosa* respectively (Fig. 1A). Our results are in accordance with previous reports, which indicate that LPS stimulation has an inhibitory effect on TLR4 gene expression (Gabler et al., 2008; Hirayama et al., 2011). This response, seeming contradictory at first, might be a self-protecting mechanism against excessive immune reactions, which may contribute to the development of endotoxin tolerance by macrophages (Hirayama et al., 2011). Moreover, our data provide evidence that, beside IRAK-4, downstream mediators of the TLR4 signaling cascade are not involved in this adaptive process.

PUFA enrichment of the culture medium did not affect gene expression of CD14, MyD88, IRAK-4 as well as TRAF6. Likewise, all PUFA tested, except DHA, failed to modulate the expression of TLR4 (Fig. 1B). DHA supplementation of the RAW264.7, however, resulted in a significant decrease in TLR4 gene expression (Fig. 1B). This effect occurred in both unstimulated and stimulated cells (Fig. 1B). To our knowledge, the action of PUFA on gene expression of downstream mediator proteins of the TLR4 pathway has not been investigated so far. Besides, conflicting results have been reported regarding the PUFA impact on TLR4 gene expression. A recently described suppressive effect of DHA, AA or fish oil on enterocytes and intestinal cells (Lu et al., 2007; Liu et al., 2012) could not been confirmed in adipose stem cells as well as HEK cells (Hsueh et al., 2011; Murumalla et al., 2012). Taking this into account and considering the data presented here it seems unlikely that modulation of gene expression of the TLR4 receptor or it downstream mediators is the driving force behind the observed inhibitory action of PUFA on LPS-stimulated NF*κ*B activation.

### PUFA inhibit the stimulation-induced recruitment of TLR4 and CD14 into membrane rafts

The membrane microdomain localization of TLR4 and CD14 was assessed by fluorescence microscopic analysis of receptor co-localization with the lipid raft marker ganglioside GM1 (Harder et al., 1998).

**Figure 2:**
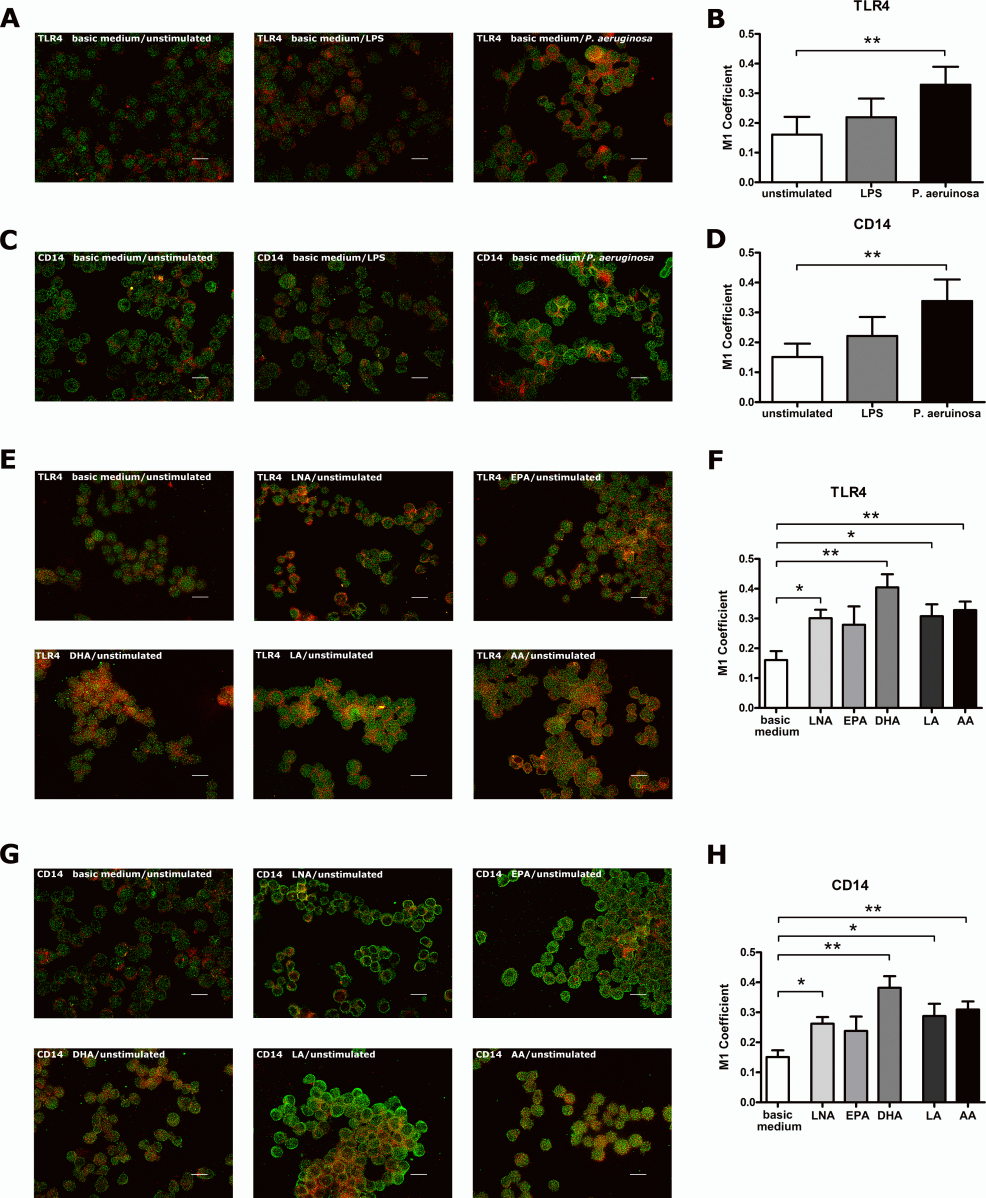
Stimulus- or PUFA-mediated modulation of the co-localization of TLR4 or CD14 with the raft marker GM1 on RAW264.7 macrophages. Co-localization of TLR4 or CD14 with the raft marker GM1 on RAW264.7 macrophages was analyzed by indirect immunofluorescence microscopy. Cells were cultured in basic medium (RPMI 1640 containing 4.5 g/L glucose, 5% v/v FCS, 0.2% v/v ethanol). Stimulation was performed by supplementation of basic medium with LPS (1 µg/mL) or viable *P. aeruginosa* (MOI 1) for 24 h. PUFA enrichment was performed by supplementation of basic medium with alpha-linolenic acid (LNA), eicosapentaenoic acid (EPA), docosahexaenoic acid (DHA), linoleic acid (LA) or arachidonic acid (AA) in a concentration of 15 µM for 72 h. Cells, grown on coverslips, were fixed in 4% paraformaldehyde, permeabilized in 0.1% Triton X-100 and subsequently immunostained using specific primary antibodies against TLR4, CD14 and GM1, respectively, as well as Alexa Fluor-labeled secondary antibodies. Representative images from 4 independent experiments were captured. GM1 is labeled in red; TLR4 and CD14 are labeled in green. Scale bar represents 20 µm. Quantification of TLR4 or CD14 co-localization with GM1 was performed by calculating the Manders‘ (M1) coefficient using the JACoP plugin of ImageJ. Data are expressed as mean ± S.D. (*N* = 4, *n* = 3). Asterisks indicate a statistically significant difference compared with the unstimulated or unsupplemented controls (^∗^*p* < 0.05, ^∗∗^*p* < 0.01). (A) Representative images showing the stimulus effects on TLR4-GM1 co-localization. High resolution images can be found in Figs. S2–S4. (B) Graph showing the stimulus effects on TLR4-GM1 co-localization. (C) Representative images showing the stimulus effects on CD14-GM1 co-localization. High resolution images can be found in Figs. S5–S7. (D) Graph showing the stimulus effects on CD14-GM1 co-localization. (E) Representative images showing the PUFA effects on TLR4-GM1 co-localization. High resolution images can be found in Figs. S8–S13. (F) Graph showing the PUFA effects on TLR4-GM1 co-localization. (G) Representative images showing the PUFA effects on CD14-GM1 co-localization. High resolution images can be found in Figs. S14–S19. (H) Graph showing the PUFA effects on CD14-GM1 co-localization.

Stimulation of RAW264.7 cultured in basic medium with LPS or viable *P. aeruginosa* resulted in an increased co-localization of both TLR4 (Figs. 2A and 2B; Figs. S2–S4) and CD14 (Figs. 2C and 2D; Figs. S5–S7) with GM1. Receptor recruitment into membrane rafts was slightly enhanced following LPS stimulation (Figs. 2B and 2D), but elevated significantly, when viable *P. aeruginosa* were used as stimulus (Figs. 2B and 2D). It is well known that the stimulation-induced interaction of TLR4 with its co-receptor CD14 takes place in lipid rafts (Olsson & Sundler, 2006; Triantafilou et al., 2006; Parker et al., 2008; Wong et al., 2009; Fessler & Parks, 2011). The selective concentration of receptors in these specialized membrane domains is believed to facilitate the assembly of the signaling complex (Nicolau et al., 2006; Fessler & Parks, 2011). In fact, investigations on model membranes have shown that the sequestration and accumulation of signal pathway components into membrane rafts increases the probability of collisions between particular proteins (Nicolau et al., 2006; Fessler & Parks, 2011). Consequently and in accordance to previous studies (Olsson & Sundler, 2006; Wong et al., 2009) the stimulation-mediated localization of TLR4 and CD14 within lipid rafts described here may represent a prerequisite for activation of the signaling cascade. Furthermore, it can be concluded from our data that complex biological stimuli, such as viable *P. aeruginosa*, are more potent activators of the TLR4 signaling cascade than purified biological molecules, such as LPS.

PUFA supplementation of unstimulated RAW264.7 resulted in a significant increased co-localization of both TLR4 (Figs. 2E and 2F; Figs. S8–S13) and CD14 (Figs. 2G and 2H; Figs. S14–S19) with GM1 with DHA having the most pronounced effect. To our knowledge this is the first study investigating the impact of PUFA on TLR4 and CD14 membrane microdomain localization on unstimulated macrophages. Our data suggest that the modulation of membrane lipid composition by n-3 or n-6 fatty acids is sufficient to alter membrane receptor distribution even in absence of particular stimulants.

**Figure 3:**
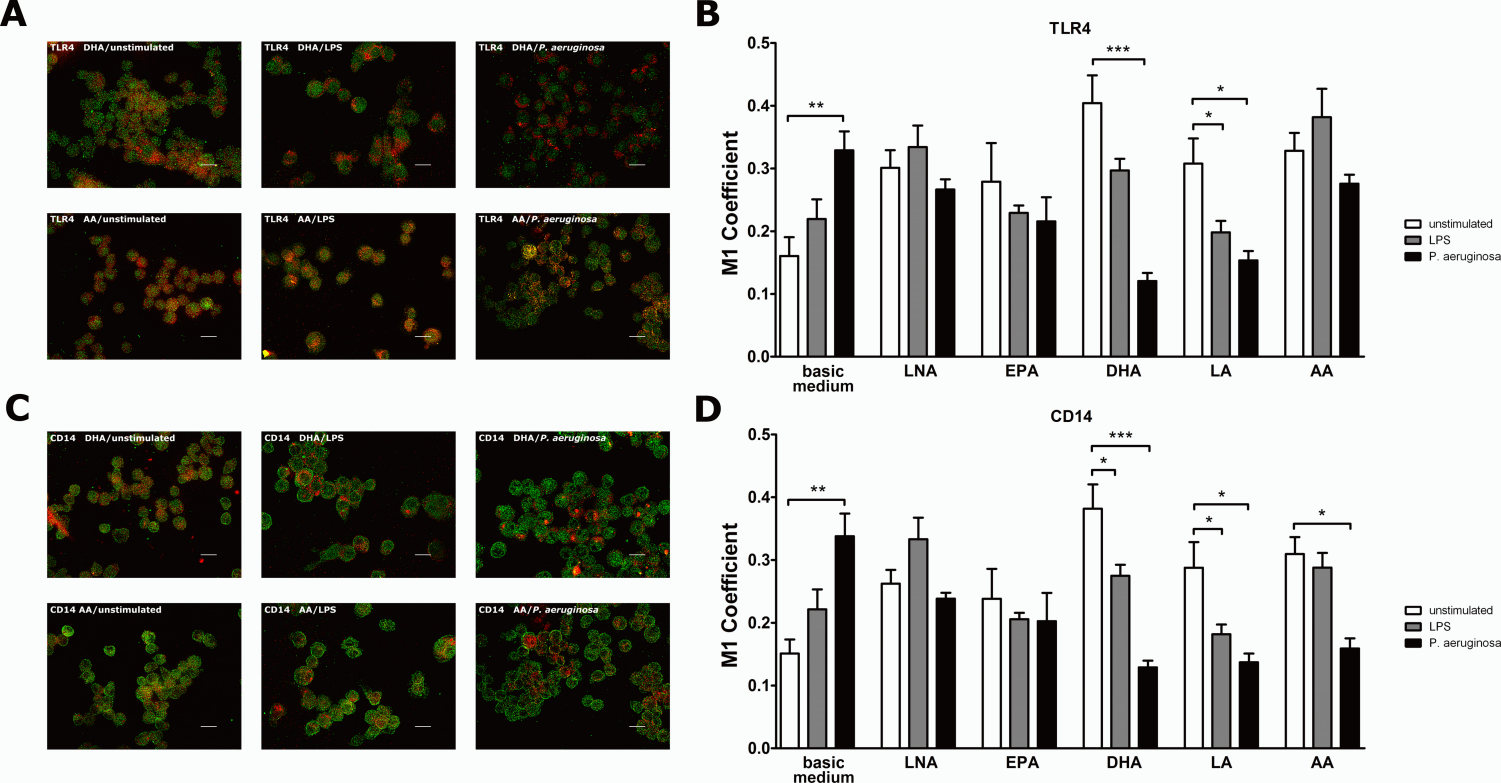
Co-localization of TLR4 or CD14 with the raft marker GM1 on PUFA-enriched and stimulated RAW264.7 macrophages. Co-localization of TLR4 or CD14 with the raft marker GM1 on RAW264.7 macrophages was analyzed by indirect immunofluorescence microscopy. Cells were cultured in basic medium (RPMI 1640 containing 4.5 g/L glucose, 5% v/v FCS, 0.2% v/v ethanol) supplemented with alpha-linolenic acid (LNA), eicosapentaenoic acid (EPA), docosahexaenoic acid (DHA), linoleic acid (LA) or arachidonic acid (AA) in a concentration of 15 µM for 72 h. Cell stimulation was performed by addition of LPS (1 µg/mL) or viable *P. aeruginosa* (MOI 1) to the culture medium in the last 24 h of incubation. Cells grown on coverslips were fixed in 4% paraformaldehyde, permeabilized in 0.1% Triton X-100 and subsequently immunostained using specific primary antibodies against TLR4, CD14 and GM1, respectively, as well as Alexa Fluor-labeled secondary antibodies. Representative images from 4 independent experiments were captured. GM1 is labeled in red; TLR4 and CD14 are labeled in green. Scale bar represents 20 µm. Quantification of TLR4 or CD14 co-localization with GM1 was performed by calculating the Manders‘ (M1) coefficient using the JACoP plugin of ImageJ. Data are expressed as mean ± S.D. (*N* = 4, *n* = 3). Asterisks indicate a statistically significant difference compared with the unstimulated controls (^∗^*p* < 0.05, ^∗∗^*p* < 0.01, ^∗∗∗^*p* < 0.001). (A) Representative images showing the stimulus effects on TLR4-GM1 co-localization of PUFA-supplemented RAW264.7. High resolution images can be found in Figs. S20–S25. (B) Graph showing the stimulus effects on TLR4-GM1 co-localization of PUFA-supplemented RAW264.7. (C) Representative images showing the stimulus effects on CD14-GM1 co-localization of PUFA-supplemented RAW264.7. High resolution images can be found in Figs. S26–S31. (D) Graph showing the stimulus effects on CD14-GM1 co-localization of PUFA-supplemented RAW264.7.

In contrast to macrophages cultured in basic medium, PUFA-supplemented RAW264.7 treated with either LPS or viable *P. aeruginosa* did not show enhanced co-localization levels of TLR4 (Figs. 3A and 3B; Figs. S20–S25) or CD14 (Figs. 3C and 3D; Figs. S26–S31) with GM1. Instead, a significant decrease in the co-localization of both TLR4 (Fig. 3B) and CD14 (Fig. 3D) with GM1 could partly be observed. A significant reduction in the co-localization of TLR4 with GM1 was observed for LA-supplemented RAW264.7 stimulated with LPS as well as for DHA- or LA-supplemented RAW264.7 stimulated with viable *P. aeruginosa* (Fig. 3B). The same applies for the co-localization of CD14 with GM1 in DHA- or LA-supplemented RAW264.7 stimulated with LPS or in DHA-, LA- or AA-supplemented RAW264.7 stimulated with viable *P. aeruginosa* (Fig. 3D). Altogether, our data indicate that the enrichment of the macrophage plasma membrane with unsaturated fatty acids abolishes the stimulation-induced clustering of TLR4 and CD14 in lipid rafts. Apparently the changes in the physical-chemical properties of the membrane microdomains going along with PUFA supplementation (Wassall & Stillwell, 2009; Turk & Chapkin, 2013) disturb the segregation of the receptors between raft and non-raft membrane domains. Since receptor sequestration in lipid rafts raises the possibility of intermolecular collisions it may be assumed that an impaired TLR4 and CD14 clustering within the microdomains interferes with receptor interaction and consequently prevents the initiation of the signaling cascade in stimulated cells.

**Figure 4:**
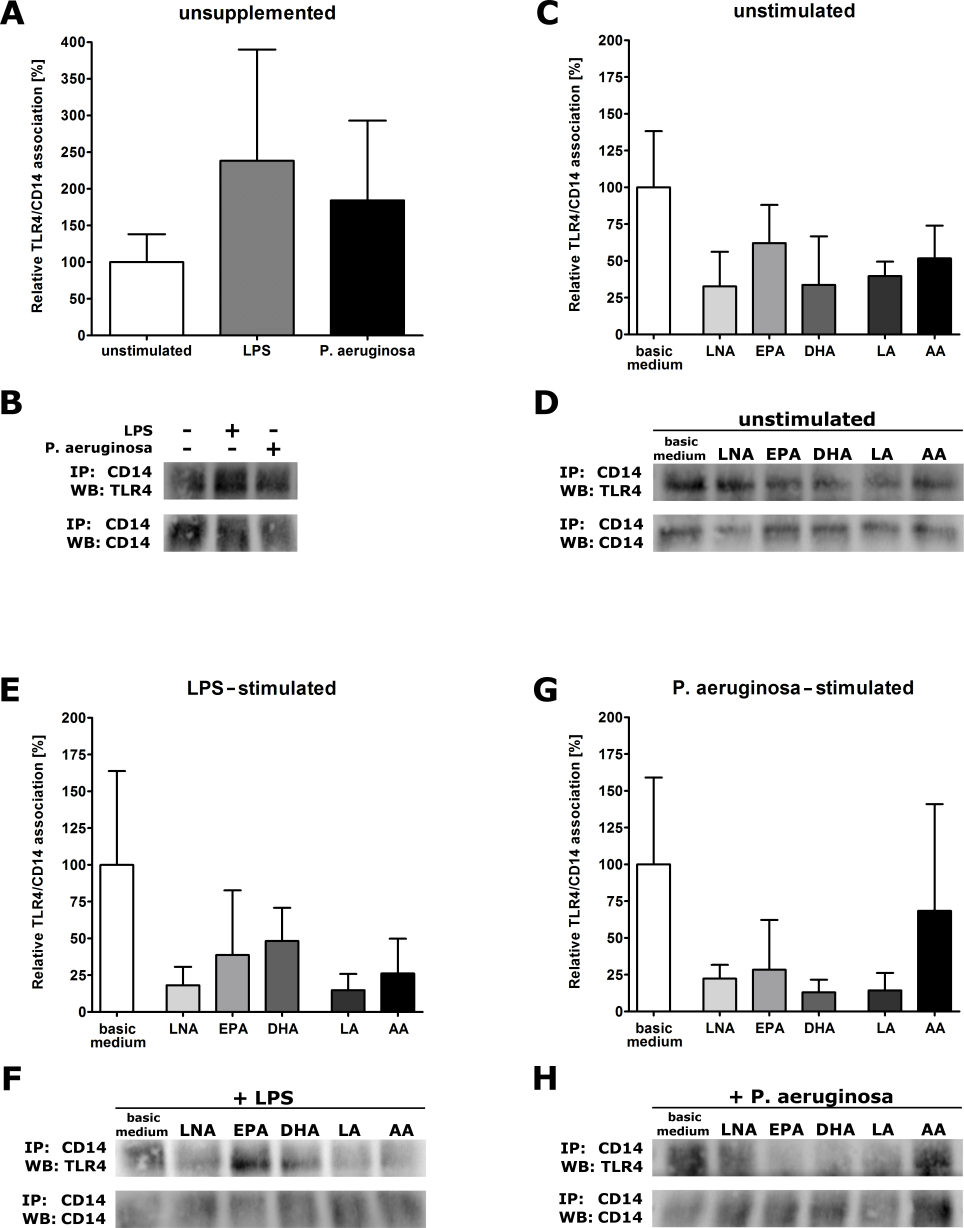
Receptor association of TLR4 and CD14 of PUFA-enriched and stimulated RAW264.7 macrophages. Formation of TLR4-CD14 complexes was determined by co-immunoprecipitation (IP) and visualized by Western blot (WB). Cells were cultured in basic medium (RPMI 1640 containing 4.5 g/L glucose, 5% v/v FCS, 0.2% v/v ethanol) supplemented with alpha-linolenic acid (LNA), eicosapentaenoic acid (EPA), docosahexaenoic acid (DHA), linoleic acid (LA) or arachidonic acid (AA) in a concentration of 15 µM for 72 h. Cell stimulation was performed by addition of LPS (1 µg/mL) or viable *P. aeruginosa* (MOI 1) to the culture medium in the last 24 h of incubation. TLR4-CD14 complexes from cell lysates were co-immunoprecipitated using an appropriate anti-mouse CD14 antibody and immunoblotted using an appropriate anti-mouse TLR4 antibody. Band size was checked using a protein ladder, and band intensities were analyzed densiometrically. The IP:CD14/WB:CD14 blot represents the lower part of the IP membrane and serves as loading control used for data normalization. To account for inter-assay variability, the stimulation/supplementation conditions compared were run on the same gel. Data from unstimulated (A) or unsupplemented (C+E+G) cells were set 100%, and values from stimulated (A) or supplemented (C+E+G) cells were expressed relative to this control. Four independent experiments were performed in duplicate for each combination of PUFA supplementation and stimulation of cells (*N* = 4, *n* = 2). (A) Graph showing the stimulus effects on TLR4-CD14 association of unsupplemented RAW264.7. (B) Representative Western blots showing the stimulus effects on TLR4-CD14 association of unsupplemented RAW264.7. (C) Graph showing the PUFA effects on TLR4-CD14 association of unstimulated RAW264.7. (D) Representative Western blots showing the PUFA effects on TLR4-CD14 association of unstimulated RAW264.7. (E) Graph showing the PUFA effects on TLR4-CD14 association of LPS-stimulated RAW264.7. (F) Representative Western blots showing the PUFA effects on TLR4-CD14 association of LPS-stimulated RAW264.7. (G) Graph showing the PUFA effects on TLR4-CD14 association of *P. aeruginosa*-stimulated RAW264.7. (H) Representative Western blots showing the PUFA effects on TLR4-CD14 association of *P. aeruginosa*-stimulated RAW264.7.

In a previous experiment it has already been shown that the n-3 PUFA DHA inhibits the stimulation induced recruitment of TLR4 into lipid rafts (Wong et al., 2009). As an extension to this study, we show that this effect does not only apply to DHA but also to various PUFA from both the n-3 and the n-6 family.

It is important to note that the Manders’ (M1) coefficient used for analyzing the microscopic data is not sensitive to differences in pixel intensities of an image (Manders, Verbeek & Aten, 1993). For this reason, we are convinced that the co-localization coefficient reflects biological reality and is not affected by differences in cellular receptor expression.

### PUFA abate the stimulation-induced TLR4 receptor complex formation

The interaction of TLR4 with its co-receptor CD14 being a prerequisite for the initiation of the TLR4 signaling cascade (McIsaac, Stadnyk & Lin, 2012), was determined using a co-immunoprecipitation assay.

There was a clear trend towards an increased association of TLR4 with CD14 due to stimulation (Figs. 4A and 4B). Our findings are in accordance with previous studies reporting TLR4-CD14 complex formation after LPS stimulation (Parker et al., 2008; Pablo Nicola et al., 2009). The importance of CD14 for LPS recognition and the initiation of the TLR4 pathway is underlined by the fact, that cells expressing TLR4 but lacking CD14 are not LPS responsive (Pablo Nicola et al., 2009).

PUFA supplementation of RAW264.7 resulted in distinct effects on TLR4-CD14 interaction. For unstimulated cells enriched with PUFA there was only a slight decrease in TLR4-CD14 association (Figs. 4C and 4D). However, stimulation-induced TLR4-CD14 complex formation was mostly attenuated by PUFA supplementation. After LPS stimulation TLR4-CD14 interaction was abated by LNA, LA and AA (Figs. 4E and 4F). After stimulation with *P. aeruginosa* all PUFA tested with the exception of AA were found to interfere with receptor-co-receptor formation (Figs. 4G and 4H). Our data support findings from a previous study performed by (Lee et al., 2003) showing that DHA-induced suppression of NF*κ*B activation is mediated by interfering with the TLR4 receptor rather than downstream effectors such as MyD88. Our results are also in line with other results of this group demonstrating that the addition of a lipid raft inhibitor to RAW264.7 prevents the LPS-mediated initiation of the TLR4 signaling cascade (Wong et al., 2009). The authors claimed that the inhibitory target of unsaturated fatty acids is the TLR4 receptor itself or its associated molecules and not downstream mediators of the signaling cascade (Lee et al., 2003; Wong et al., 2009). In addition, they supposed that the absence of a cellular response to LPS stimulation in presence of unsaturated fatty acids may be due to an inhibition of raft formation going along with a reduction in interactions of TLR4 receptor complex components (Wong et al., 2009). Our results clearly confirm this hypothesis thereby providing first evidence of an impaired TLR4 receptor complex formation by PUFA enrichment of macrophage lipid rafts. Of note, in our experimental setting PUFA other than DHA seem to be even more effective in inhibiting stimulation-induced TLR4-CD14 association. The fatty acid family (n-3 versus n-6), in any case, appears to be less important for the observed effects of PUFA supplementation on macrophage TLR4 receptor complex formation.

## Conclusions

Several studies show the central importance of membrane raft lipid remodeling for cellular signaling (Fessler & Parks, 2011). Lipid rafts, under certain biophysical conditions, serve as dynamic platforms for the assembly of specific proteins thereby facilitating receptor complex formation. However, as shown by our results, slight changes in membrane lipid composition, which modulate the physical-chemical properties of rafts (Schumann et al., 2011), are sufficient to induce a reordering of membrane domains thereby leading to altered cellular signal transduction. Hence, microdomain disruption via macrophage PUFA supplementation has a potential as non-toxic strategy to impede inflammatory signaling.

## Supplemental Information

10.7717/peerj.1663/supp-1 Figure S1 Stimulus- and/or PUFA-mediated modulation of IL-1*β*, IL-6 and TNF-*α* synthesis of RAW264.7 macrophages RAW264.7 macrophages were cultured in basic medium (RPMI 1640 containing 4.5 g/L glucose, 5% v/v FCS, 0.2% v/v ethanol). The pro-inflammatory cytokines IL-1*β*, IL-6 and TNF-*α* were quantified in cell supernatants by ELISA. (A) Cytokine production of cells stimulated with LPS (1 µg/mL) for 24 h. (B) Cytokine production of cells supplemented with alpha-linolenic acid (LNA), eicosapentaenoic acid (EPA), docosahexaenoic acid (DHA), linoleic acid (LA) or arachidonic acid (AA) in a concentration of 15 µM for 72 h. (C) Cytokine production of cells supplemented with alpha-linolenic acid (LNA), eicosapentaenoic acid (EPA), docosahexaenoic acid (DHA), linoleic acid (LA) or arachidonic acid (AA) in a concentration of 15 µM for 72 h, and stimulated with LPS (1 µg/mL) in the last 24 h of supplementation. Data are mean ± S.D. (*N* = 6). Asterisks indicate a statistically significant difference compared with the unstimulated or unsupplemented controls (^∗^*p* < 0.05, ^∗∗^*p* < 0.01, ^∗∗∗^*p* < 0.001). For further details please refer to Schoeniger et al. (2011).

10.7717/peerj.1663/supp-2 Figure S2 High resolution microscopic image showing TLR4-GM1 co-localization of unstimulated RAW264.7 macrophages cultured in basic medium GM1 is labeled in red; TLR4 is labeled in green. Scale bar represents 20 µm. Related to Fig. 2A.

10.7717/peerj.1663/supp-3 Figure S3 High resolution microscopic image showing TLR4-GM1 co-localization of LPS-stimulated (1 µg/mL, 24 h) RAW264.7 macrophages cultured in basic medium GM1 is labeled in red; TLR4 is labeled in green. Scale bar represents 20 µm. Related to Fig. 2A.

10.7717/peerj.1663/supp-4 Figure S4 High resolution microscopic image showing TLR4-GM1 co-localization of *P. aeruginosa*-stimulated (MOI 1, 24 h) RAW264.7 macrophages cultured in basic medium GM1 is labeled in red; TLR4 is labeled in green. Scale bar represents 20 µm. Related to Fig. 2A.

10.7717/peerj.1663/supp-5 Figure S5 High resolution microscopic image showing CD14-GM1 co-localization of unstimulated RAW264.7 macrophages cultured in basic medium GM1 is labeled in red; CD14 is labeled in green. Scale bar represents 20 µm. Related to Fig. 2C.

10.7717/peerj.1663/supp-6 Figure S6 High resolution microscopic image showing CD14-GM1 co-localization of LPS-stimulated (1 µg/mL, 24 h) RAW264.7 macrophages cultured in basic medium GM1 is labeled in red; CD14 is labeled in green. Scale bar represents 20 µm. Related to Fig. 2C.

10.7717/peerj.1663/supp-7 Figure S7 High resolution microscopic image showing CD14-GM1 co-localization of *P. aeruginosa*-stimulated (MOI 1, 24 h) RAW264.7 macrophages cultured in basic medium GM1 is labeled in red; CD14 is labeled in green. Scale bar represents 20 µm. Related to Fig. 2C.

10.7717/peerj.1663/supp-8 Figure S8 High resolution microscopic image showing TLR4-GM1 co-localization of unstimulated RAW264.7 macrophages cultured in basic medium GM1 is labeled in red; TLR4 is labeled in green. Scale bar represents 20 µm. Related to Fig. 2E.

10.7717/peerj.1663/supp-9 Figure S9 High resolution microscopic image showing TLR4-GM1 co-localization of unstimulated RAW264.7 macrophages cultured in basic medium enriched with alpha- linoleic acid (LNA; 15 µM, 72 h) GM1 is labeled in red; TLR4 is labeled in green. Scale bar represents 20 µm. Related to Fig. 2E.

10.7717/peerj.1663/supp-10 Figure S10 High resolution microscopic image showing TLR4-GM1 co-localization of unstimulated RAW264.7 macrophages cultured in basic medium enriched with eicosapentaenoic acid (EPA; 15 µM, 72) GM1 is labeled in red; TLR4 is labeled in green. Scale bar represents 20 µm. Related to Fig. 2E.

10.7717/peerj.1663/supp-11 Figure S11 High resolution microscopic image showing TLR4-GM1 co-localization of unstimulated RAW264.7 macrophages cultured in basic medium enriched with docosahexaenoic acid (DHA; 15 µM, 72 h) GM1 is labeled in red; TLR4 is labeled in green. Scale bar represents 20 µm. Related to Fig. 2E.

10.7717/peerj.1663/supp-12 Figure S12 High resolution microscopic image showing TLR4-GM1 co-localization of unstimulated RAW264.7 macrophages cultured in basic medium enriched with linoleic acid (LA; 15 µM, 72 h) GM1 is labeled in red; TLR4 is labeled in green. Scale bar represents 20 µm. Related to Fig. 2E.

10.7717/peerj.1663/supp-13 Figure S13 High resolution microscopic image showing TLR4-GM1 co-localization of unstimulated RAW264.7 macrophages cultured in basic medium enriched with arachidonic acid (AA; 15 µM, 72 h) GM1 is labeled in red; TLR4 is labeled in green. Scale bar represents 20 µm. Related to Fig. 2E.

10.7717/peerj.1663/supp-14 Figure S14 High resolution microscopic image showing CD14-GM1 co-localization of unstimulated RAW264.7 macrophages cultured in basic medium GM1 is labeled in red; CD14 is labeled in green. Scale bar represents 20 µm. Related to Fig. 2G.

10.7717/peerj.1663/supp-15 Figure S15 High resolution microscopic image showing CD14-GM1 co-localization of unstimulated RAW264.7 macrophages cultured in basic medium enriched with alpha-linoleic acid (LNA; 15 µM, 72 h) GM1 is labeled in red; CD14 is labeled in green. Scale bar represents 20 µm. Related to Fig. 2G.

10.7717/peerj.1663/supp-16 Figure S16 High resolution microscopic image showing CD14-GM1 co-localization of unstimulated RAW264.7 macrophages cultured in basic medium enriched with eicosapentaenoic acid (EPA; 15 µM, 72 h) GM1 is labeled in red; CD14 is labeled in green. Scale bar represents 20 µm. Related to Fig. 2G.

10.7717/peerj.1663/supp-17 Figure S17 High resolution microscopic image showing CD14-GM1 co-localization of unstimulated RAW264.7 macrophages cultured in basic medium enriched with docosahexaenoic acid (DHA; 15 µM, 72 h) GM1 is labeled in red; CD14 is labeled in green. Scale bar represents 20 µm. Related to Fig. 2G.

10.7717/peerj.1663/supp-18 Figure S18 High resolution microscopic image showing CD14-GM1 co-localization of unstimulated RAW264.7 macrophages cultured in basic medium enriched with linoleic acid (LA; 15 µM, 72 h) GM1 is labeled in red; CD14 is labeled in green. Scale bar represents 20 µm. Related to Fig. 2G.

10.7717/peerj.1663/supp-19 Figure S19 High resolution microscopic image showing CD14-GM1 co-localization of unstimulated RAW264.7 macrophages cultured in basic medium enriched with arachidonic acid (AA; 15 µM, 72 h) GM1 is labeled in red; CD14 is labeled in green. Scale bar represents 20 µm. Related to Fig. 2G.

10.7717/peerj.1663/supp-20 Figure S20 High resolution microscopic image showing TLR4-GM1 co-localization of unstimulated RAW264.7 cultured in basic medium enriched with docosahexaenoic acid (DHA; 15 µM, 72 h) GM1 is labeled in red; TLR4 is labeled in green. Scale bar represents 20 µm. Related to Fig. 3A.

10.7717/peerj.1663/supp-21 Figure S21 High resolution microscopic image showing TLR4-GM1 co-localization of LPS-stimulated (1 µg/mL, 24 h) RAW264.7 cultured in basic medium enriched with docosahexaenoic acid (DHA; 15 µM, 72 h) GM1 is labeled in red; TLR4 is labeled in green. Scale bar represents 20 µm. Related to Fig. 3A.

10.7717/peerj.1663/supp-22 Figure S22 High resolution microscopic image showing TLR4-GM1 co-localization of *P. aeruginosa*-stimulated (MOI 1, 24 h) RAW264.7 cultured in basic medium enriched with docosahexaenoic acid (DHA; 15 µM, 72 h) GM1 is labeled in red; TLR4 is labeled in green. Scale bar represents 20 µm. Related to Fig. 3A.

10.7717/peerj.1663/supp-23 Figure S23 High resolution microscopic image showing TLR4-GM1 co-localization of unstimulated RAW264.7 cultured in basic medium enriched with arachidonic acid (AA; 15 µM, 72 h) GM1 is labeled in red; TLR4 is labeled in green. Scale bar represents 20 µm. Related to Fig. 3A.

10.7717/peerj.1663/supp-24 Figure S24 High resolution microscopic image showing TLR4-GM1 co-localization of LPS-stimulated (1 µg/mL, 24 h) RAW264.7 cultured in basic medium enriched with arachidonic acid (AA; 15 µM, 72 h) GM1 is labeled in red; TLR4 is labeled in green. Scale bar represents 20 µm. Related to Fig. 3A.

10.7717/peerj.1663/supp-25 Figure S25 High resolution microscopic image showing TLR4-GM1 co-localization of *P. aeruginosa*-stimulated (MOI 1, 24 h) RAW264.7 cultured in basic medium enriched with arachidonic acid (AA; 15 µM, 72 h) GM1 is labeled in red; TLR4 is labeled in green. Scale bar represents 20 µm. Related to Fig. 3A.

10.7717/peerj.1663/supp-26 Figure S26 High resolution microscopic image showing CD14-GM1 co-localization of unstimulated RAW264.7 cultured in basic medium enriched with docosahexaenoic acid (DHA; 15 µM, 72 h) GM1 is labeled in red; CD14 is labeled in green. Scale bar represents 20 µm. Related to Fig. 3C.

10.7717/peerj.1663/supp-27 Figure S27 High resolution microscopic image showing CD14-GM1 co-localization of LPS-stimulated (1 µg/mL, 24 h) RAW264.7 cultured in basic medium enriched with docosahexaenoic acid (DHA; 15 µM, 72 h) GM1 is labeled in red; CD14 is labeled in green. Scale bar represents 20 µm. Related to Fig. 3C.

10.7717/peerj.1663/supp-28 Figure S28 High resolution microscopic image showing CD14-GM1 co-localization of *P. aeruginosa*-stimulated (MOI 1, 24 h) RAW264.7 cultured in basic medium enriched with docosahexaenoic acid (DHA; 15 µM, 72 h) GM1 is labeled in red; CD14 is labeled in green. Scale bar represents 20 µm. Related to Fig. 3C.

10.7717/peerj.1663/supp-29 Figure S29 High resolution microscopic image showing CD14-GM1 co-localization of unstimulated RAW264.7 cultured in basic medium enriched with arachidonic acid (AA; 15 µM, 72 h) GM1 is labeled in red; CD14 is labeled in green. Scale bar represents 20 µm. Related to Fig. 3C.

10.7717/peerj.1663/supp-30 Figure S30 High resolution microscopic image showing CD14-GM1 co-localization of LPS-stimulated (1 µg/mL, 24 h) RAW264.7 cultured in basic medium enriched with arachidonic acid (AA; 15 µM, 72 h) GM1 is labeled in red; CD14 is labeled in green. Scale bar represents 20 µm. Related to Fig. 3C.

10.7717/peerj.1663/supp-31 Figure S31 High resolution microscopic image showing CD14-GM1 co-localization of *P. aeruginosa*-stimulated (MOI 1, 24 h) RAW264.7 cultured in basic medium enriched with arachidonic acid (AA; 15 µM, 72 h) GM1 is labeled in red; CD14 is labeled in green. Scale bar represents 20 µm. Related to Fig. 3C.

## Additional Information and Declarations

### Data Availability

The following information was supplied regarding data availability: The raw data is deposited in an institutional repository of the Institute of Physiological Chemistry, Faculty of Veterinary Medicine, University of Leipzig.
