# Supplementary figures and images for: LPS- or Pseudomonas aeruginosa-mediated activation of the macrophage TLR4 signaling cascade depends on membrane lipid composition
Source: PeerJ. 2016 Feb 4;4:e1663. doi: 10.7717/peerj.1663 (PMC4748739; doi:10.7717/peerj.1663)

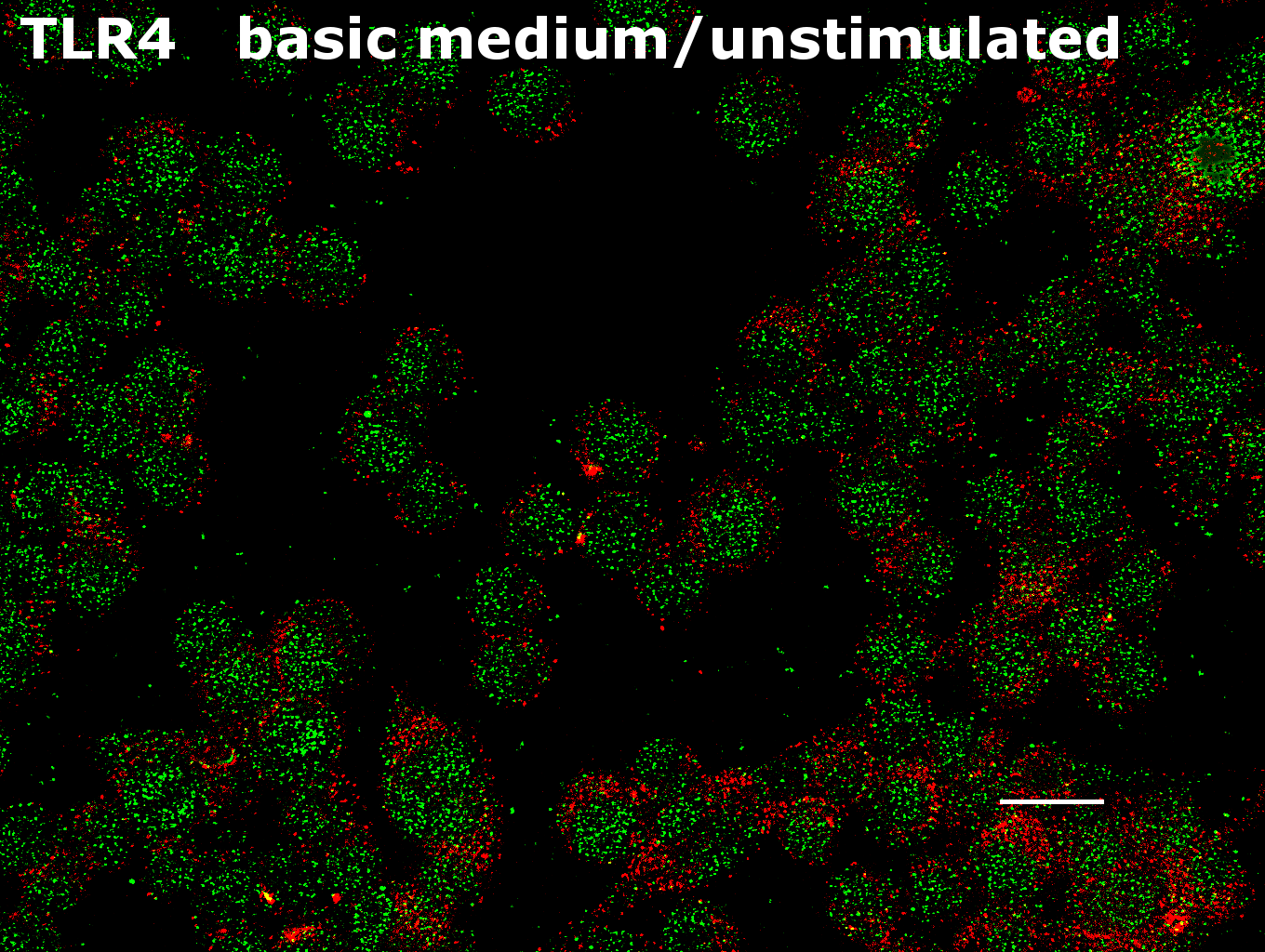

Supplement: Figure S2 — GM1 is labeled in red; TLR4 is labeled in green. Scale bar represents 20 µm. Related to Fig. 2A. [file peerj-04-1663-s002.png]

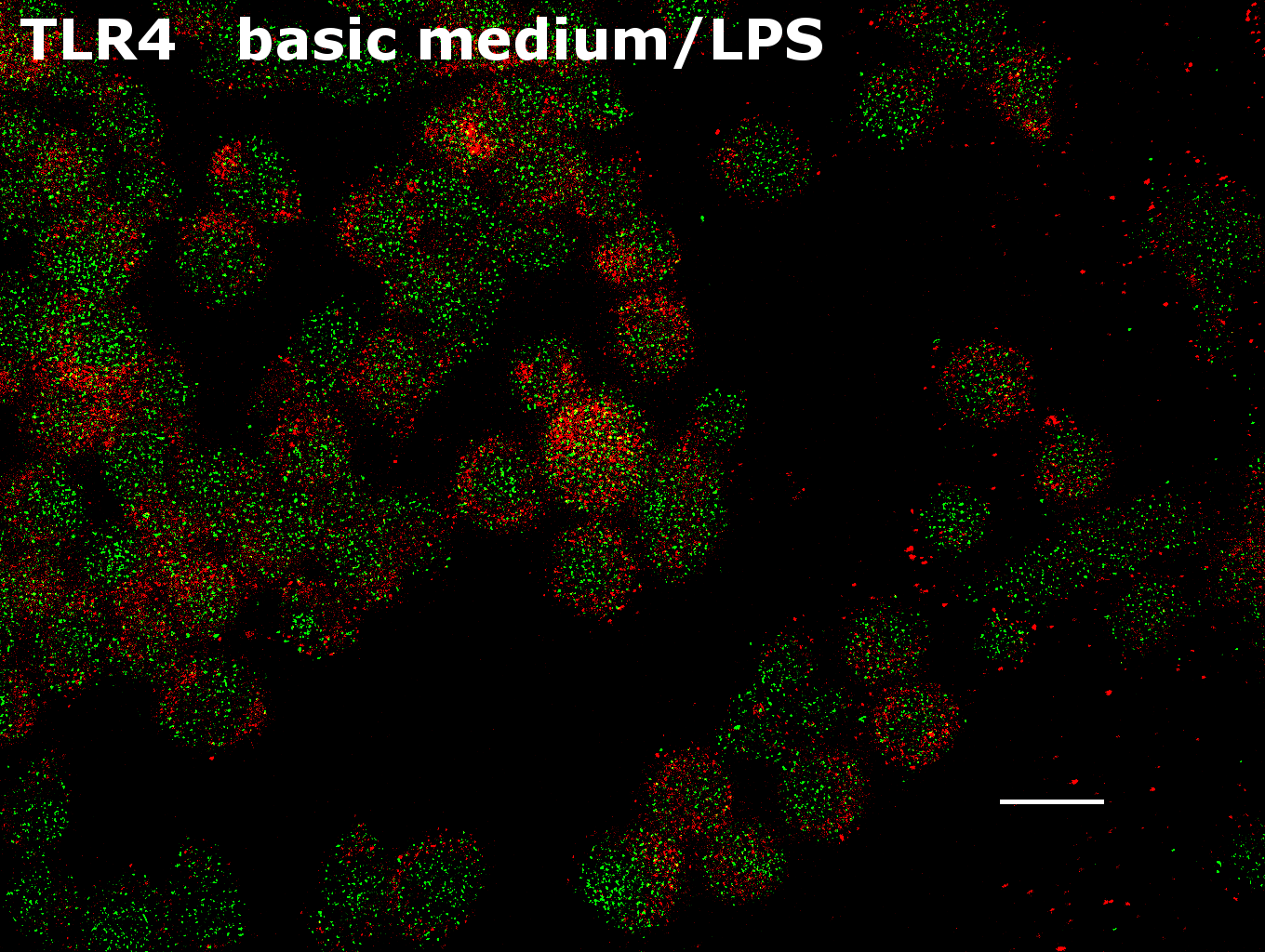

Supplement: Figure S3 — GM1 is labeled in red; TLR4 is labeled in green. Scale bar represents 20 µm. Related to Fig. 2A. [file peerj-04-1663-s003.png]

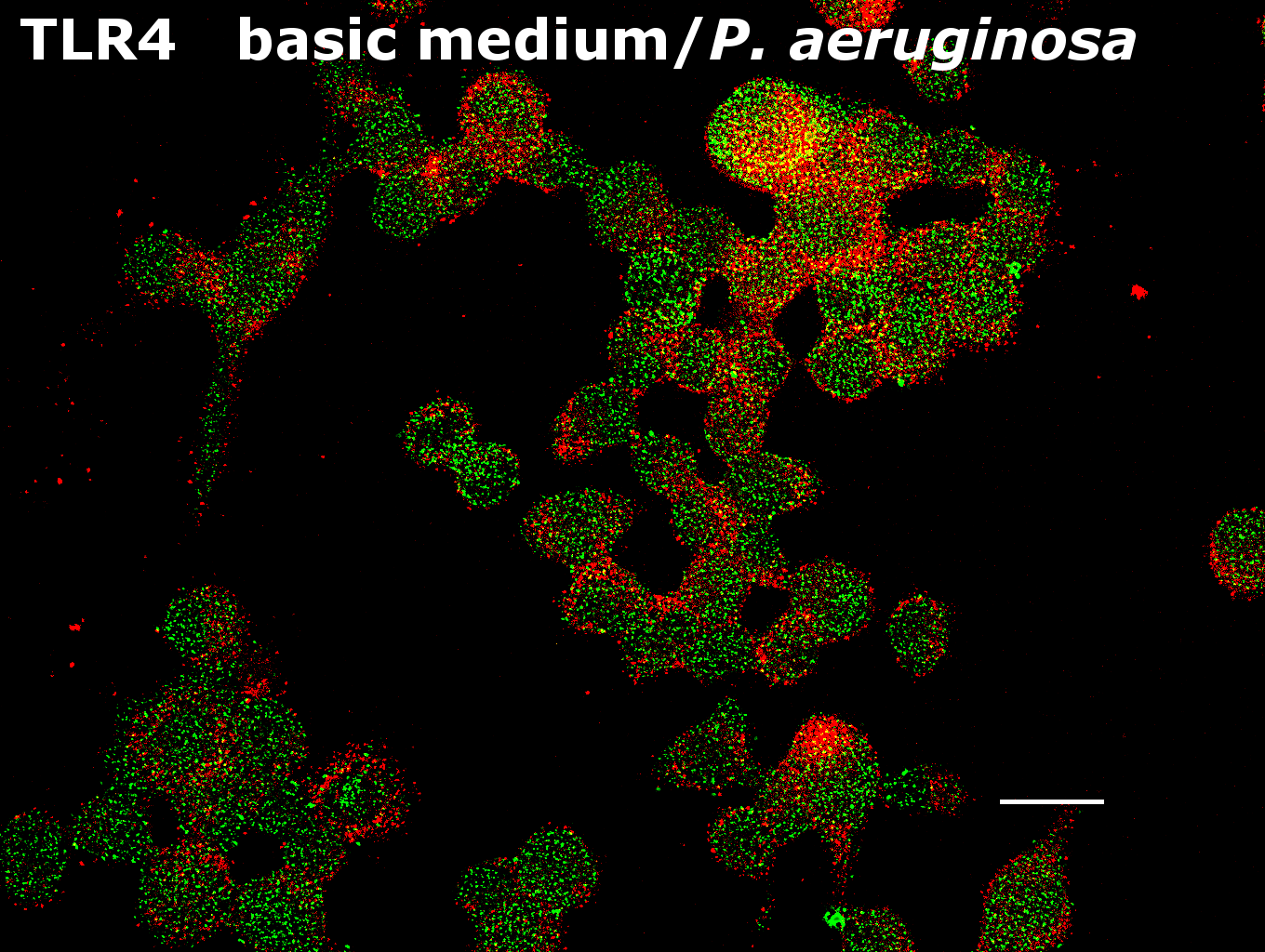

Supplement: Figure S4 — GM1 is labeled in red; TLR4 is labeled in green. Scale bar represents 20 µm. Related to Fig. 2A. [file peerj-04-1663-s004.png]

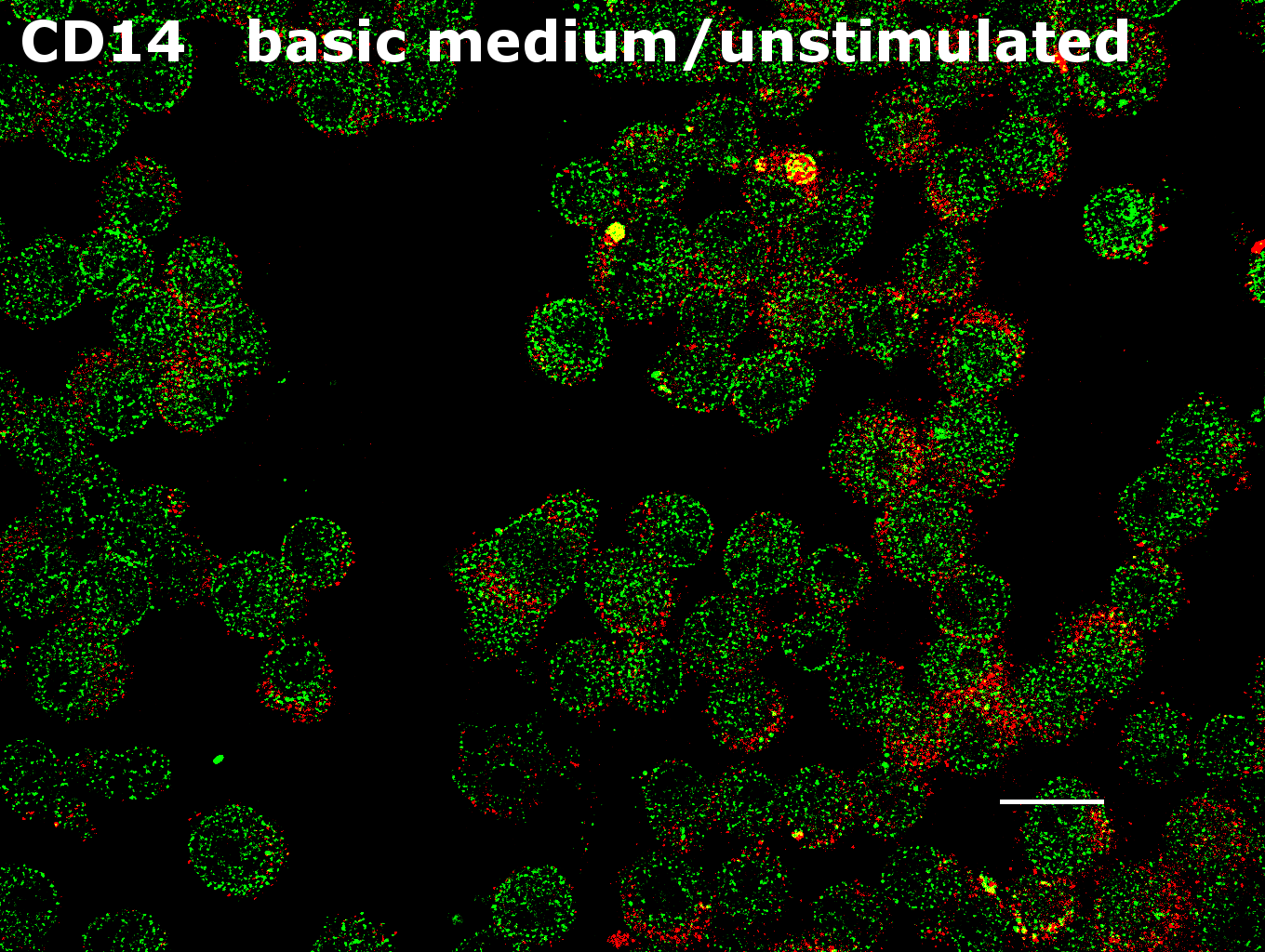

Supplement: Figure S5 — GM1 is labeled in red; CD14 is labeled in green. Scale bar represents 20 µm. Related to Fig. 2C. [file peerj-04-1663-s005.png]

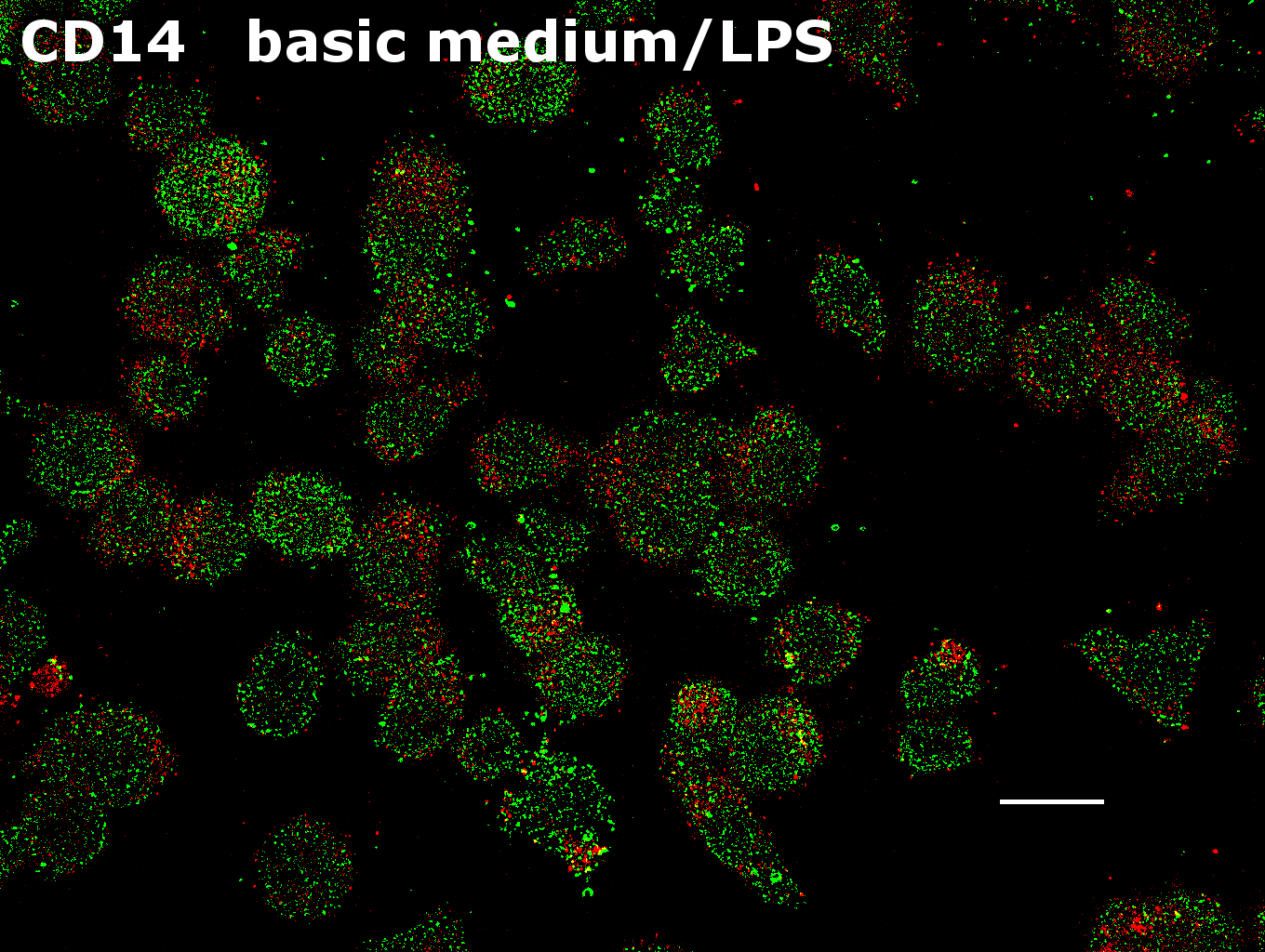

Supplement: Figure S6 — GM1 is labeled in red; CD14 is labeled in green. Scale bar represents 20 µm. Related to Fig. 2C. [file peerj-04-1663-s006.png]

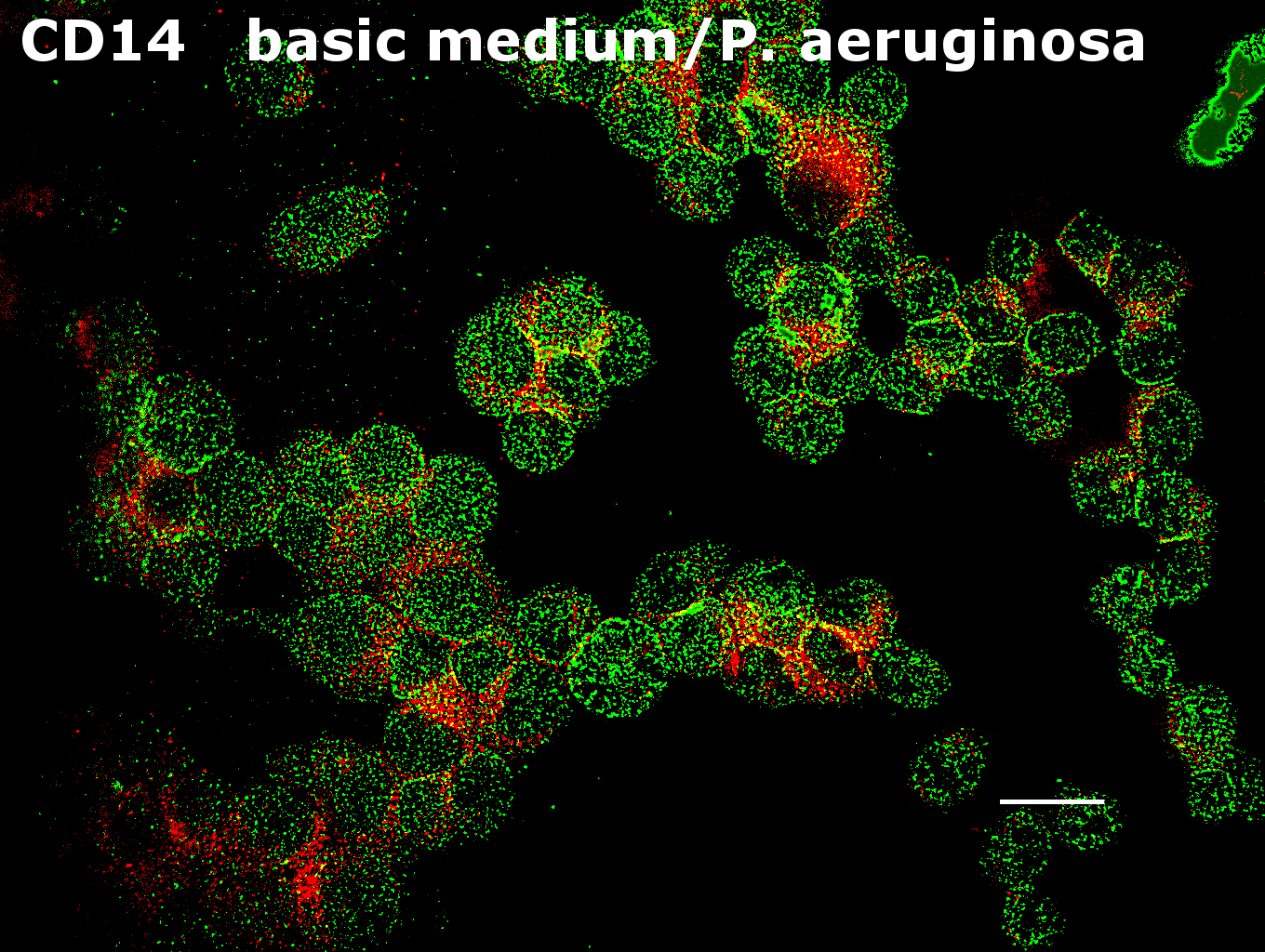

Supplement: Figure S7 — GM1 is labeled in red; CD14 is labeled in green. Scale bar represents 20 µm. Related to Fig. 2C. [file peerj-04-1663-s007.png]

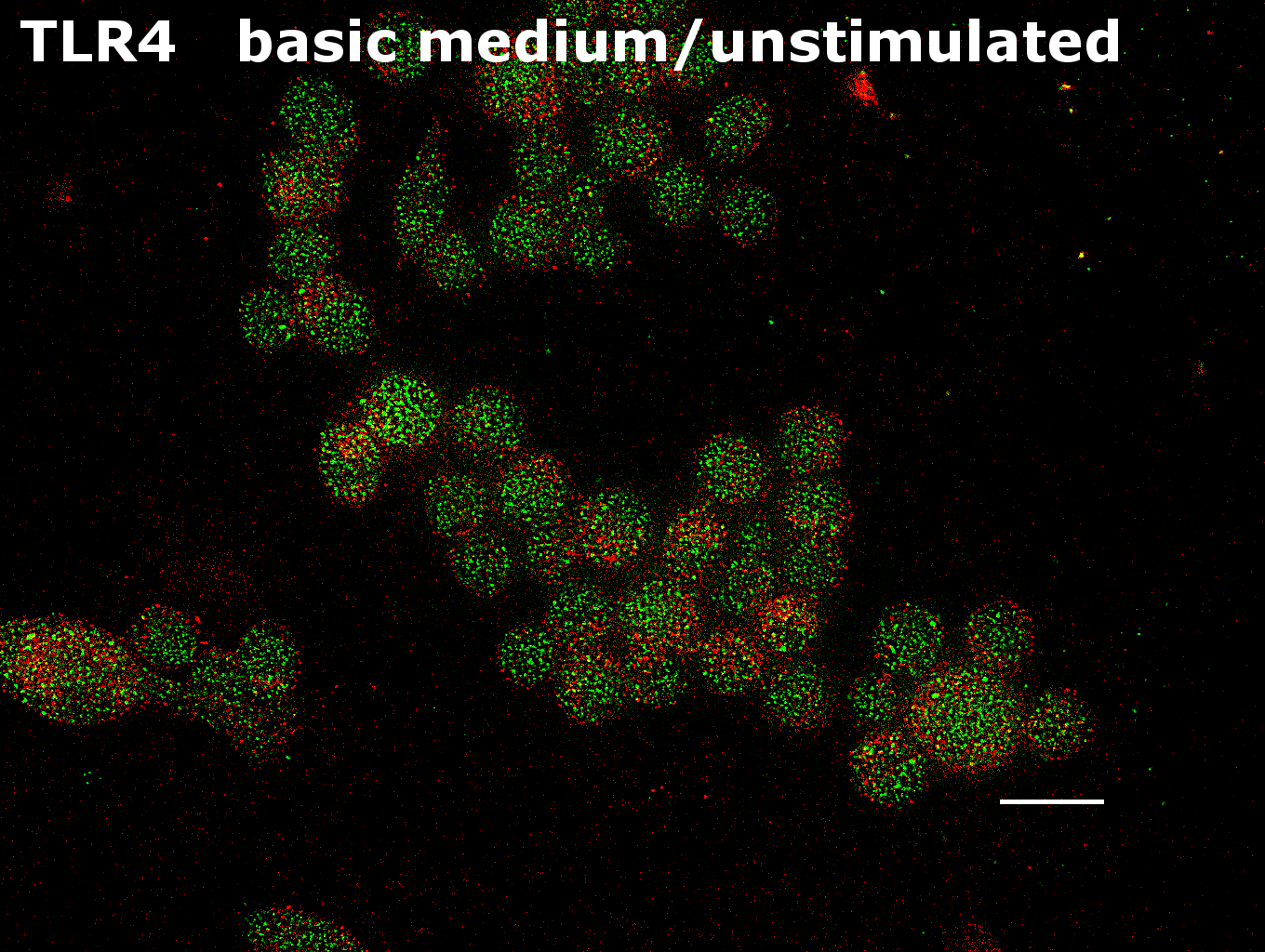

Supplement: Figure S8 — GM1 is labeled in red; TLR4 is labeled in green. Scale bar represents 20 µm. Related to Fig. 2E. [file peerj-04-1663-s008.png]

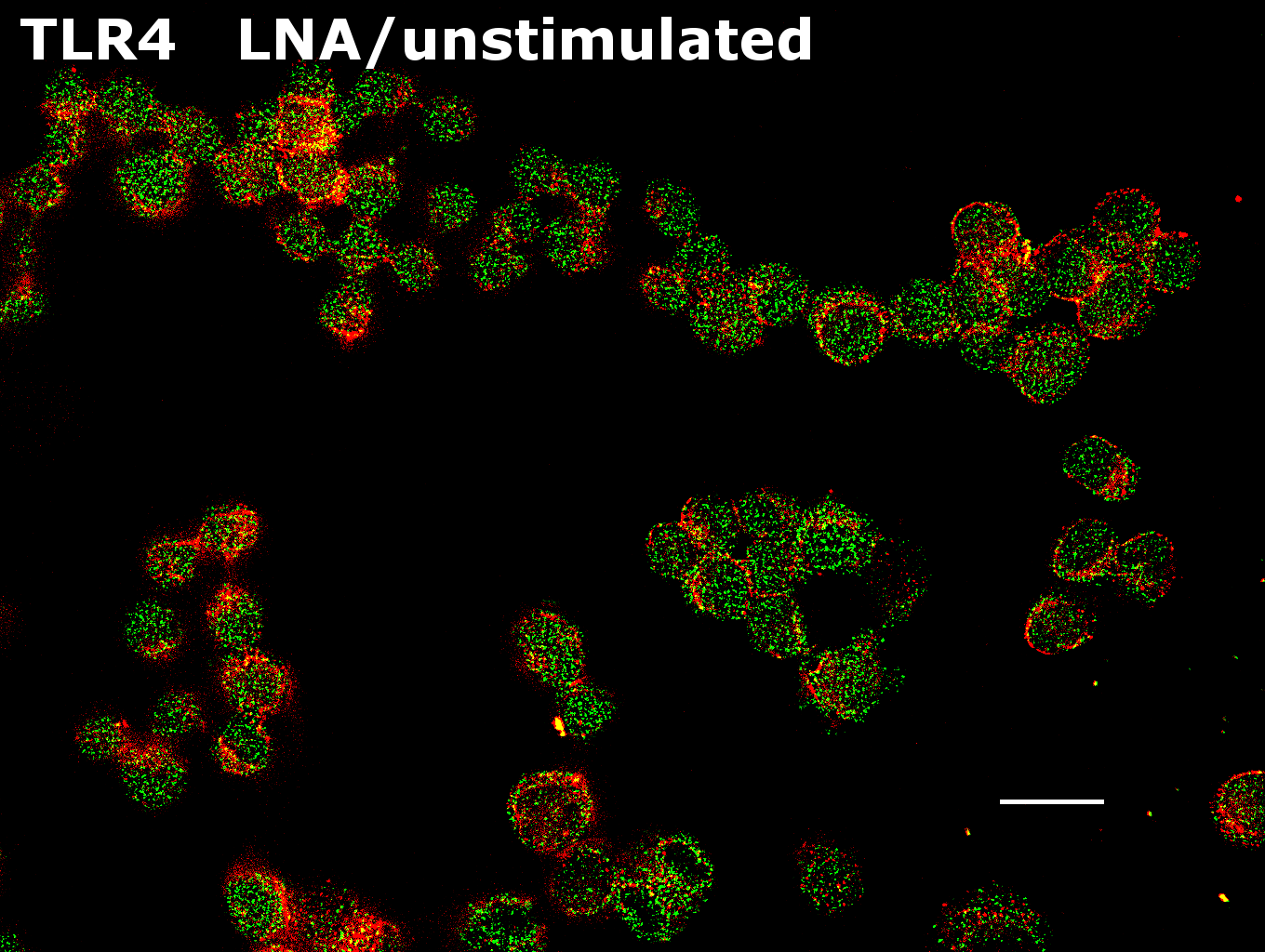

Supplement: Figure S9 — GM1 is labeled in red; TLR4 is labeled in green. Scale bar represents 20 µm. Related to Fig. 2E. [file peerj-04-1663-s009.png]

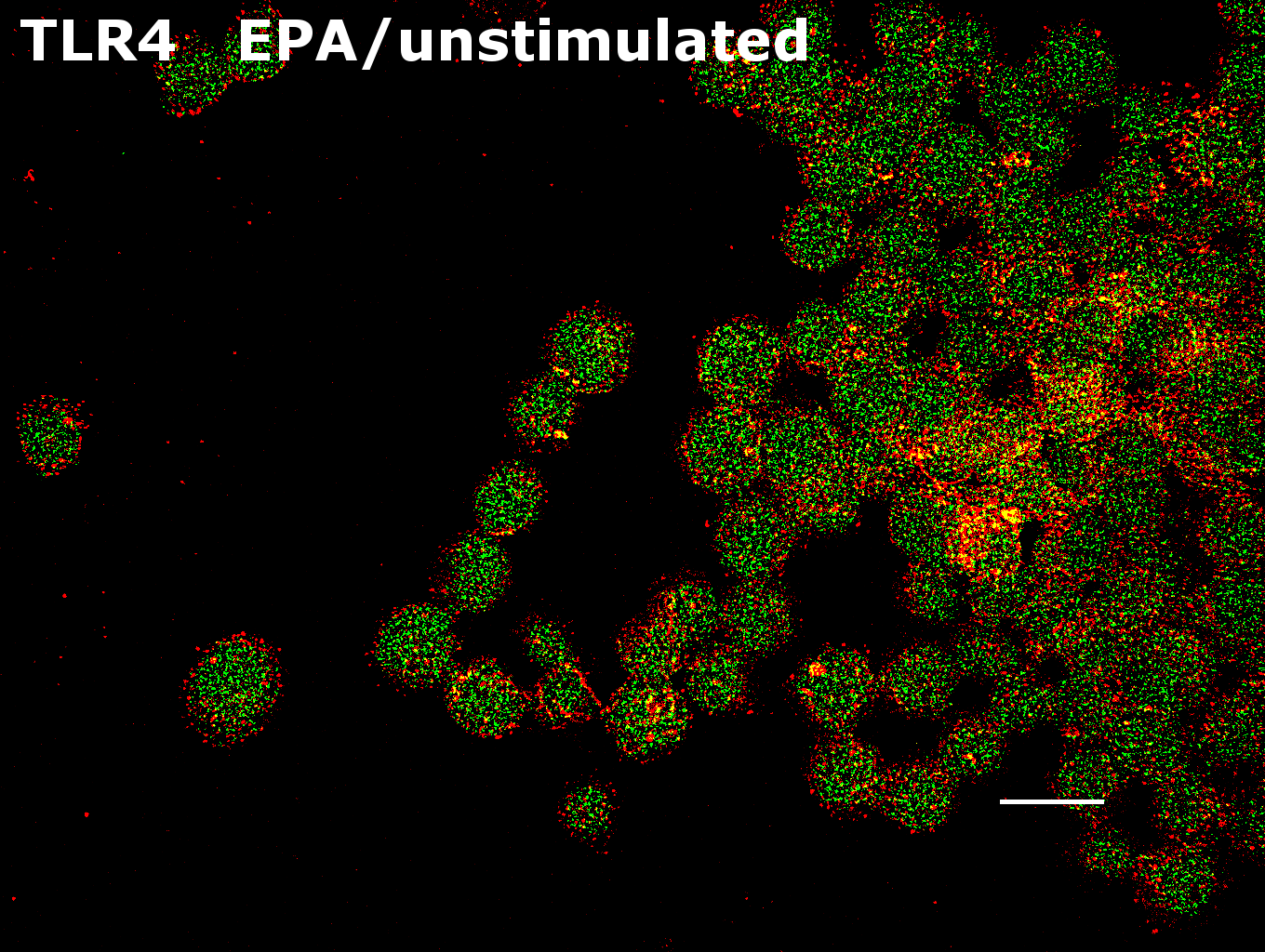

Supplement: Figure S10 — GM1 is labeled in red; TLR4 is labeled in green. Scale bar represents 20 µm. Related to Fig. 2E. [file peerj-04-1663-s010.png]

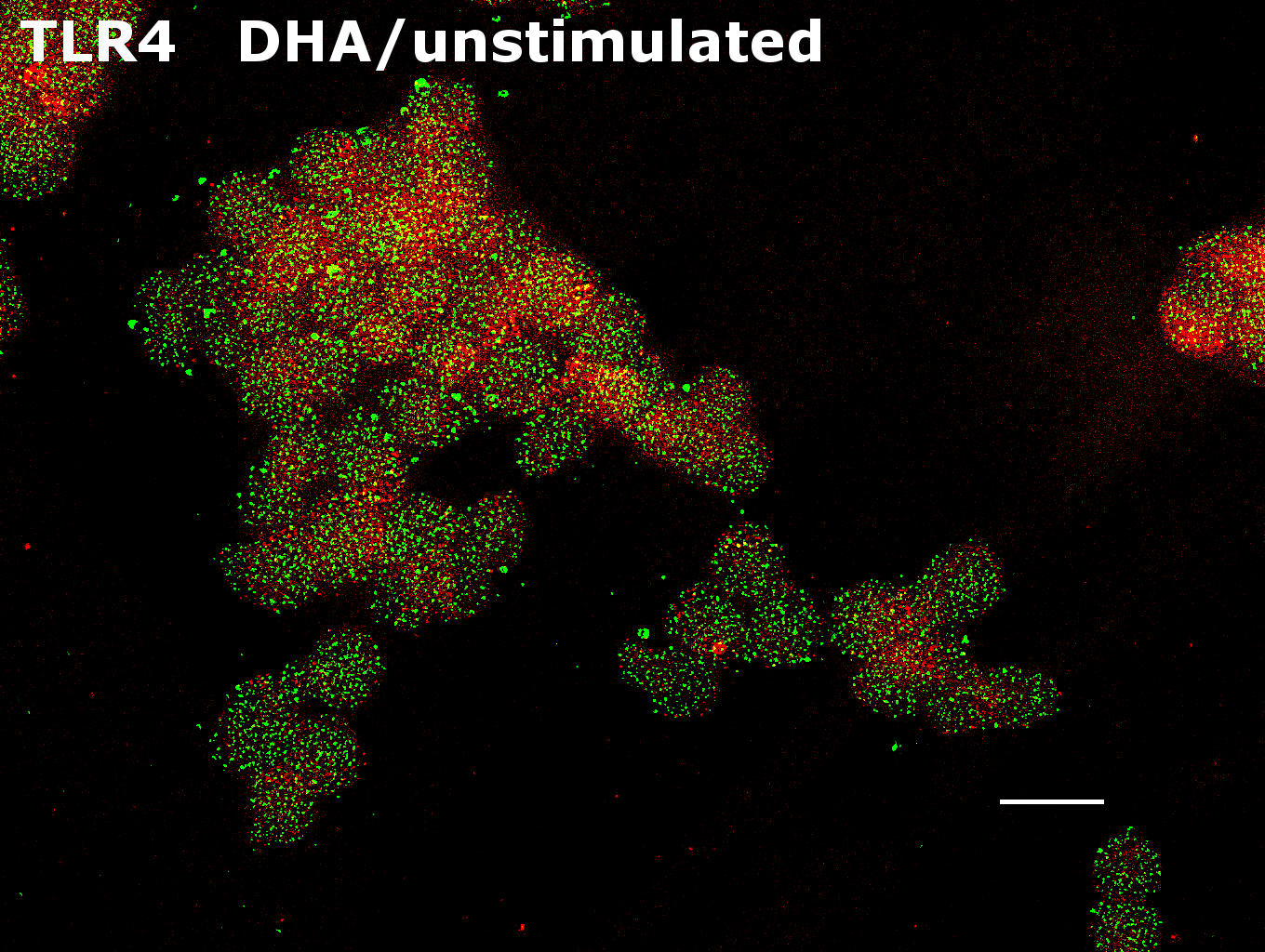

Supplement: Figure S11 — GM1 is labeled in red; TLR4 is labeled in green. Scale bar represents 20 µm. Related to Fig. 2E. [file peerj-04-1663-s011.png]

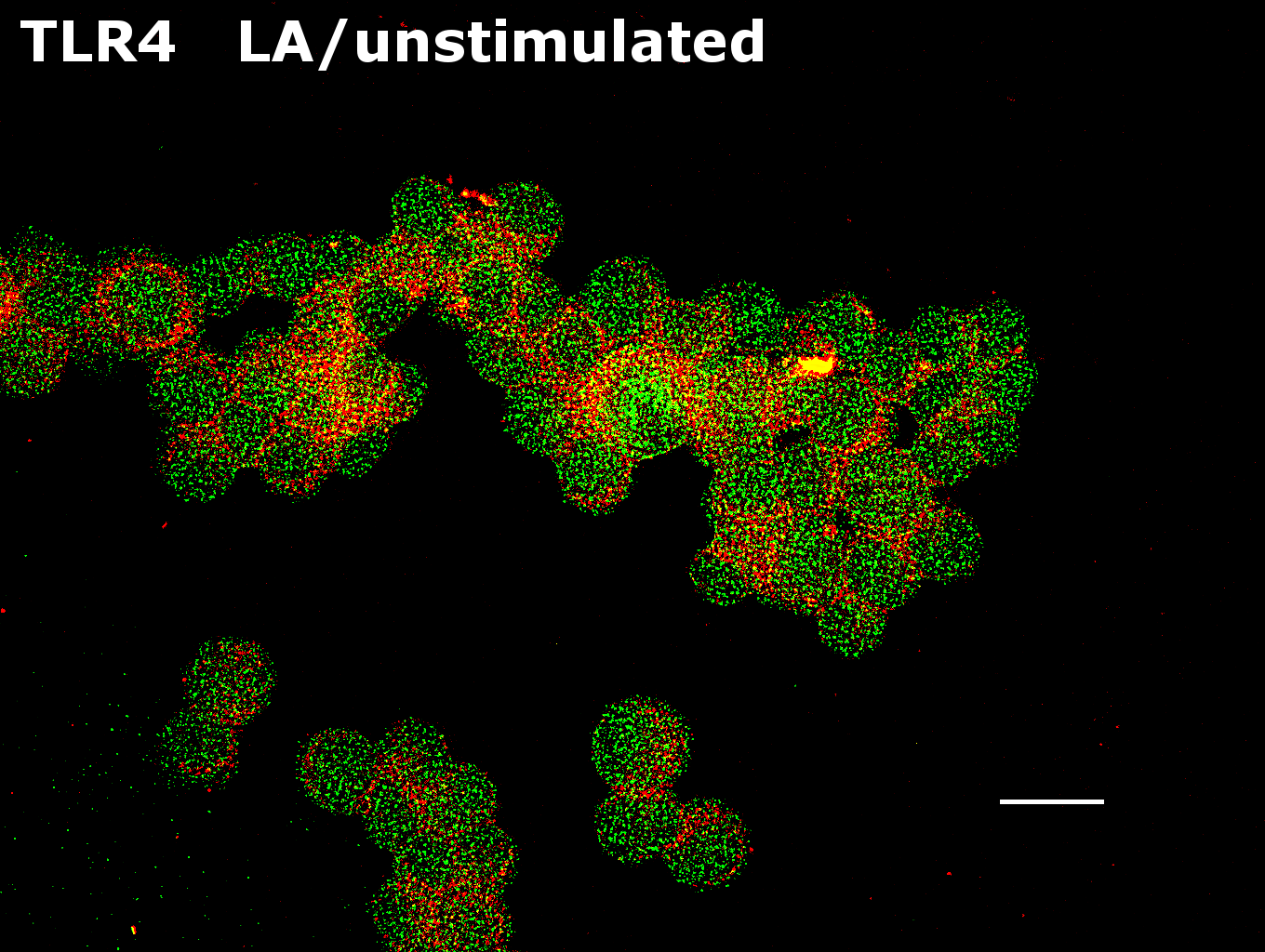

Supplement: Figure S12 — GM1 is labeled in red; TLR4 is labeled in green. Scale bar represents 20 µm. Related to Fig. 2E. [file peerj-04-1663-s012.png]

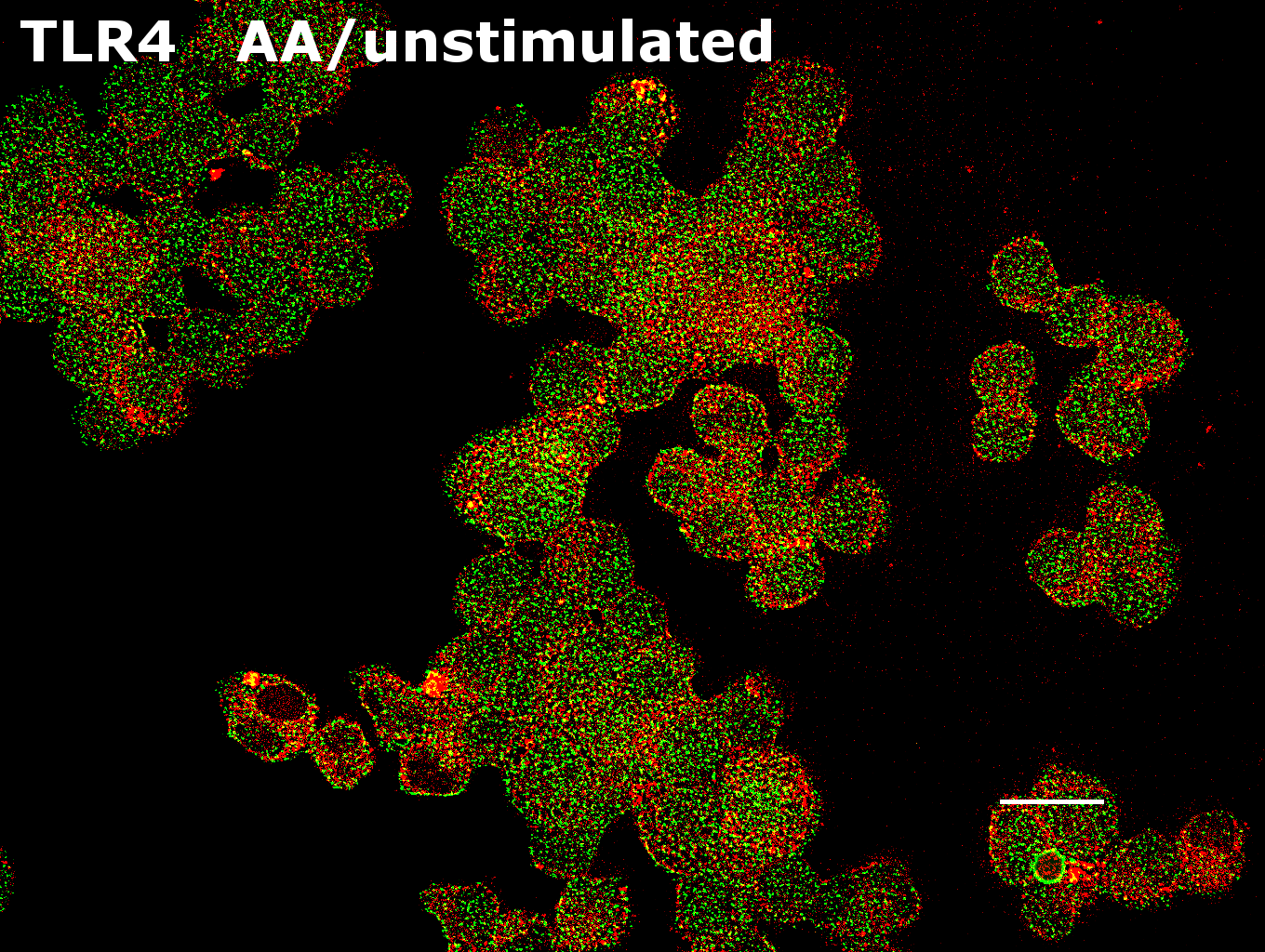

Supplement: Figure S13 — GM1 is labeled in red; TLR4 is labeled in green. Scale bar represents 20 µm. Related to Fig. 2E. [file peerj-04-1663-s013.png]

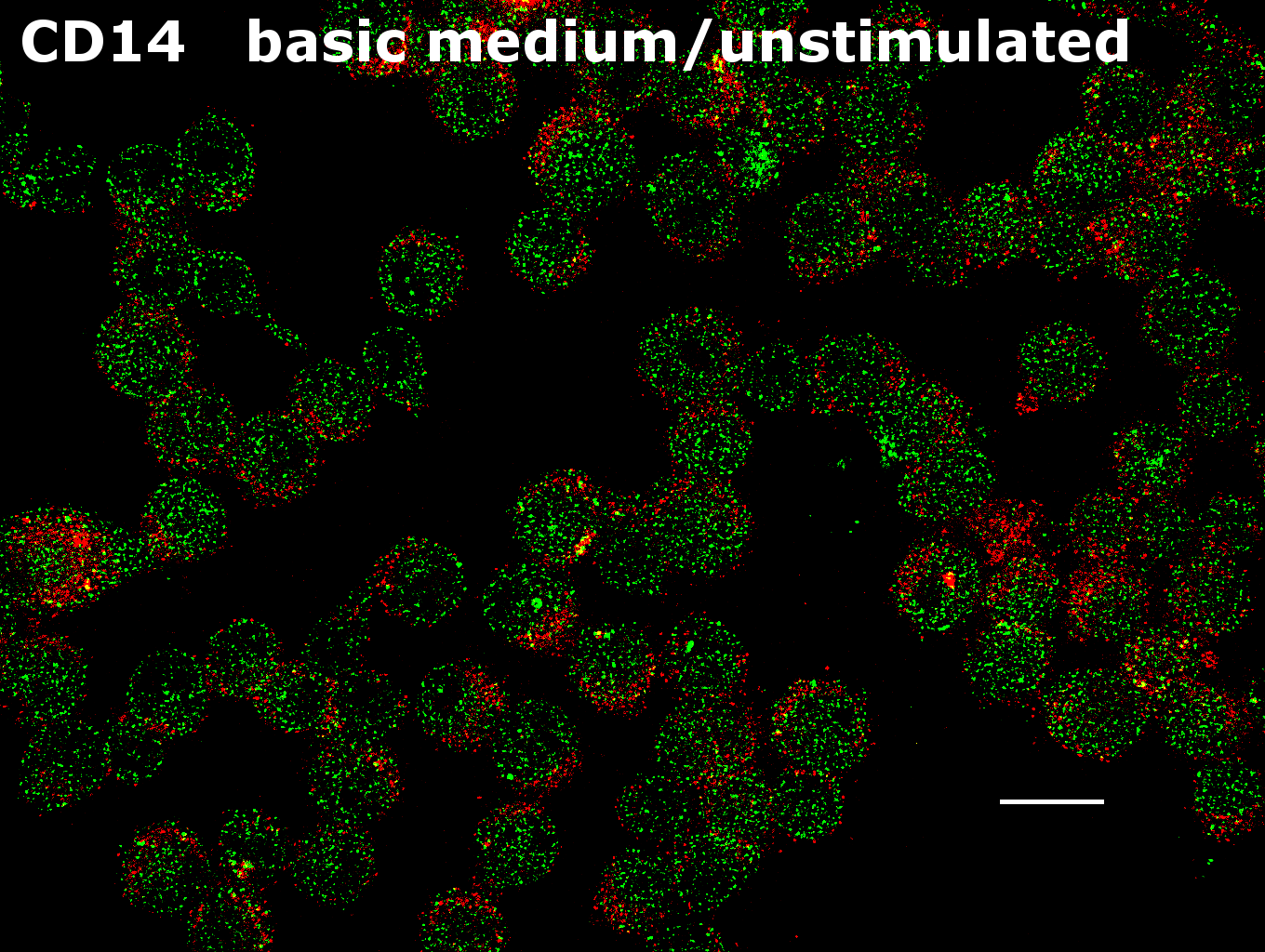

Supplement: Figure S14 — GM1 is labeled in red; CD14 is labeled in green. Scale bar represents 20 µm. Related to Fig. 2G. [file peerj-04-1663-s014.png]

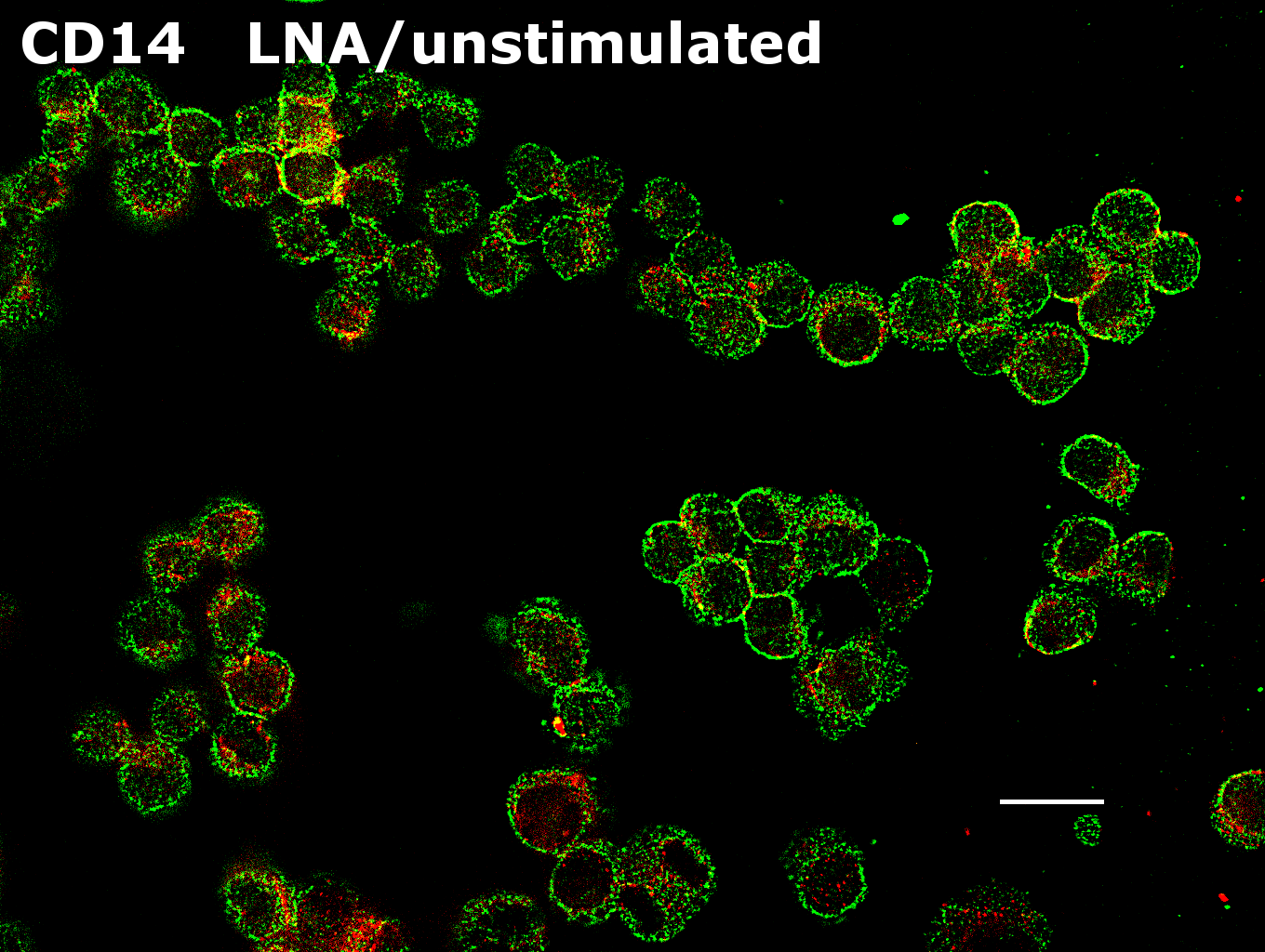

Supplement: Figure S15 — GM1 is labeled in red; CD14 is labeled in green. Scale bar represents 20 µm. Related to Fig. 2G. [file peerj-04-1663-s015.png]

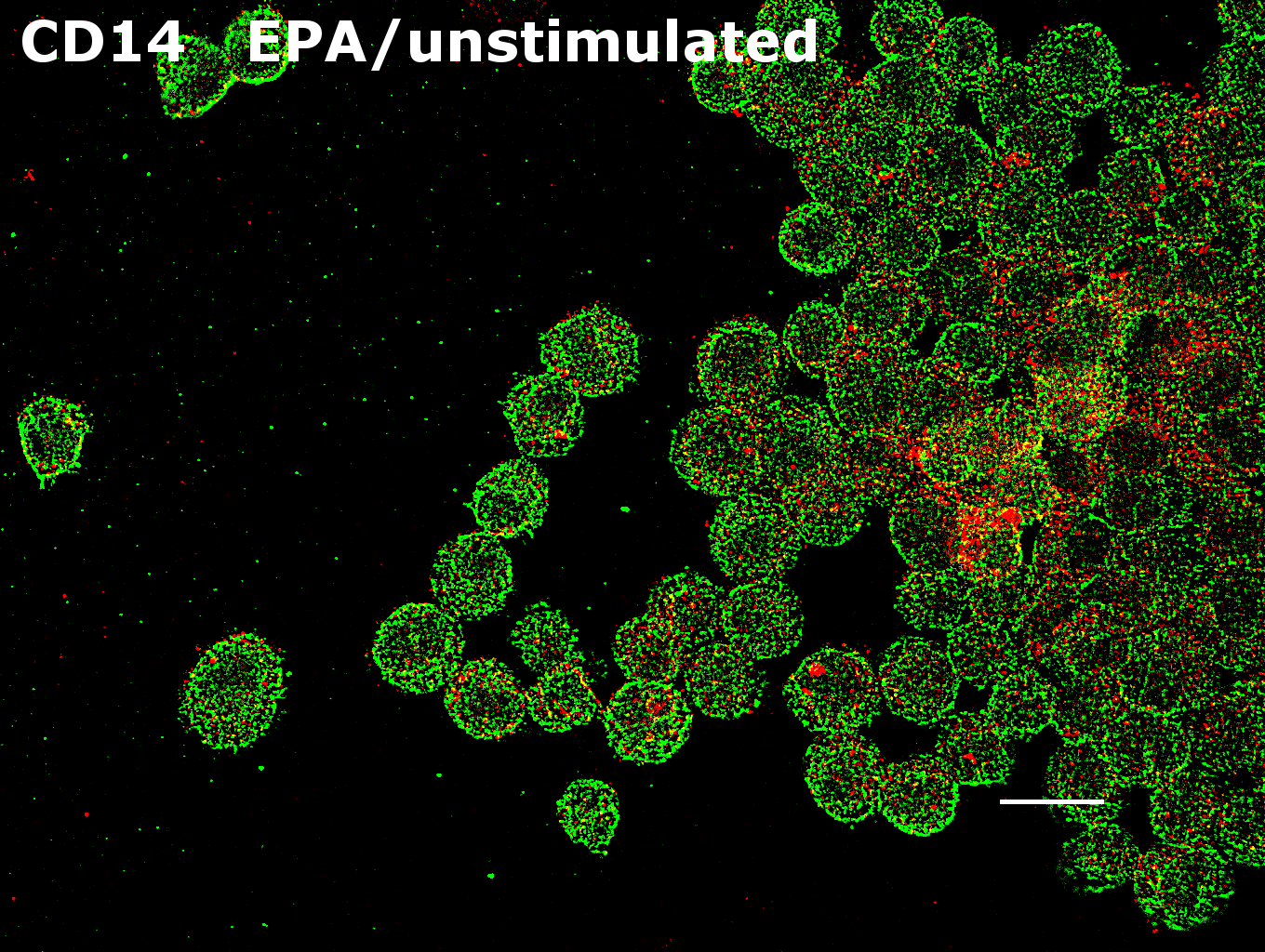

Supplement: Figure S16 — GM1 is labeled in red; CD14 is labeled in green. Scale bar represents 20 µm. Related to Fig. 2G. [file peerj-04-1663-s016.png]

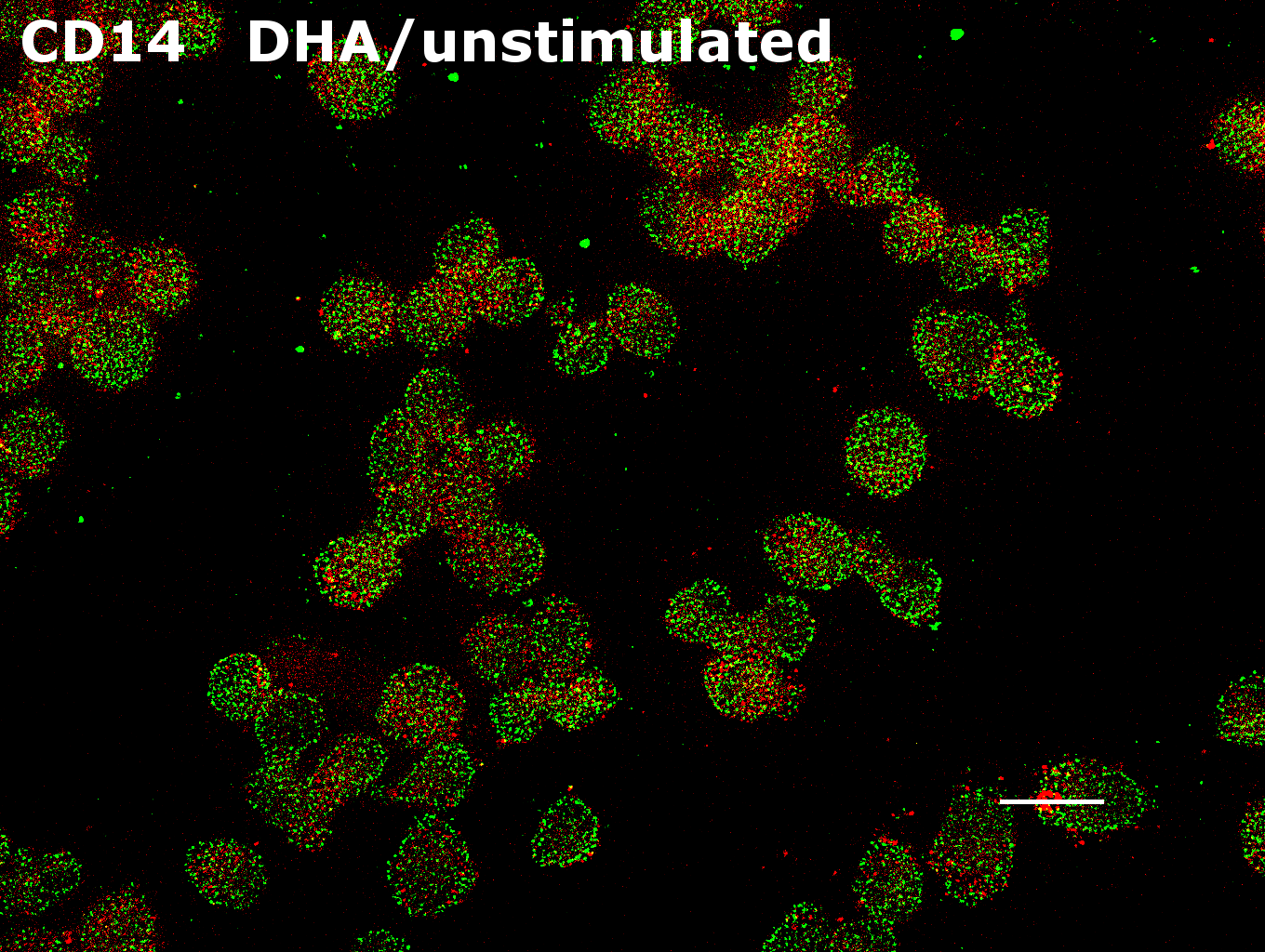

Supplement: Figure S17 — GM1 is labeled in red; CD14 is labeled in green. Scale bar represents 20 µm. Related to Fig. 2G. [file peerj-04-1663-s017.png]

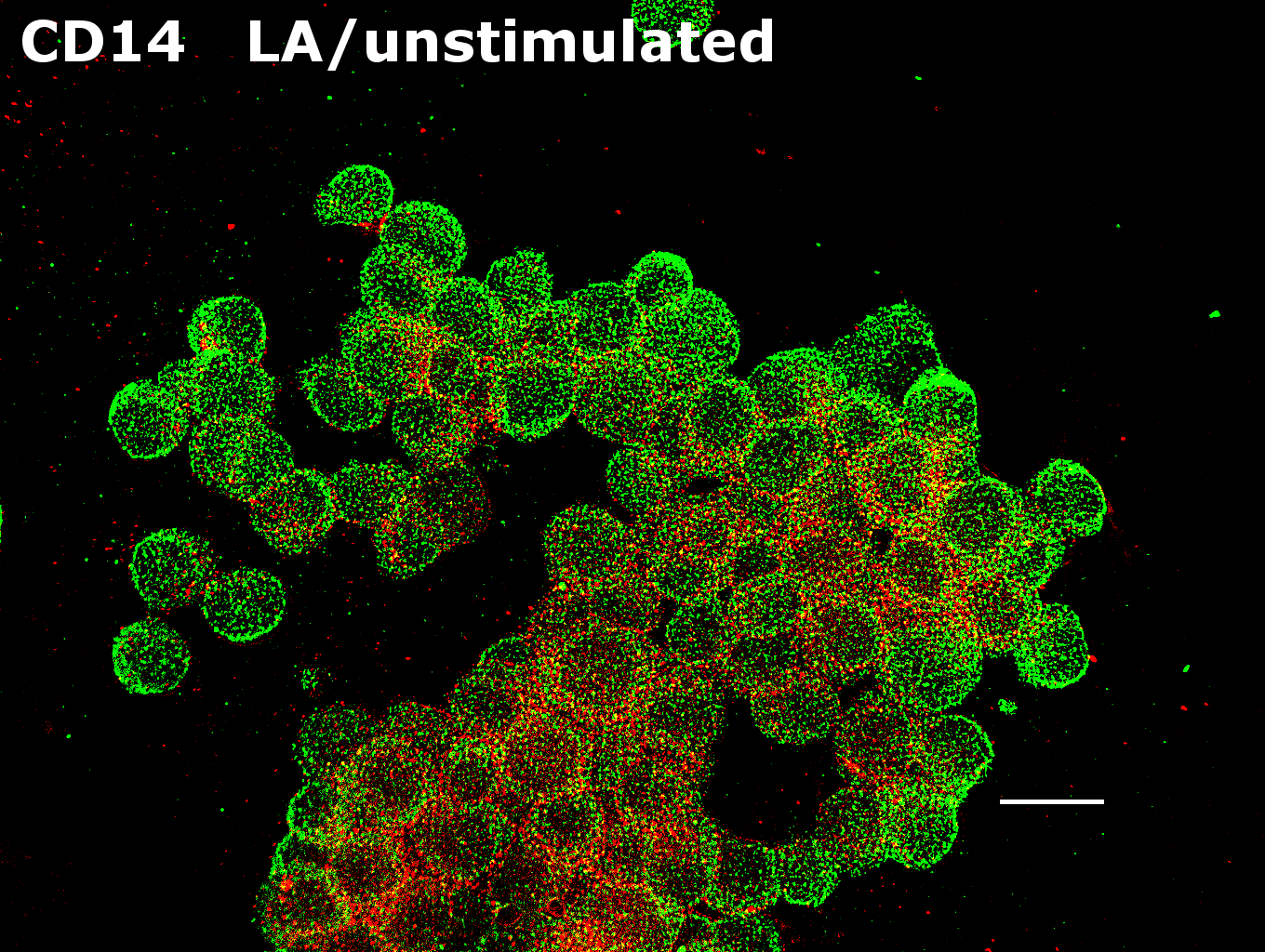

Supplement: Figure S18 — GM1 is labeled in red; CD14 is labeled in green. Scale bar represents 20 µm. Related to Fig. 2G. [file peerj-04-1663-s018.png]

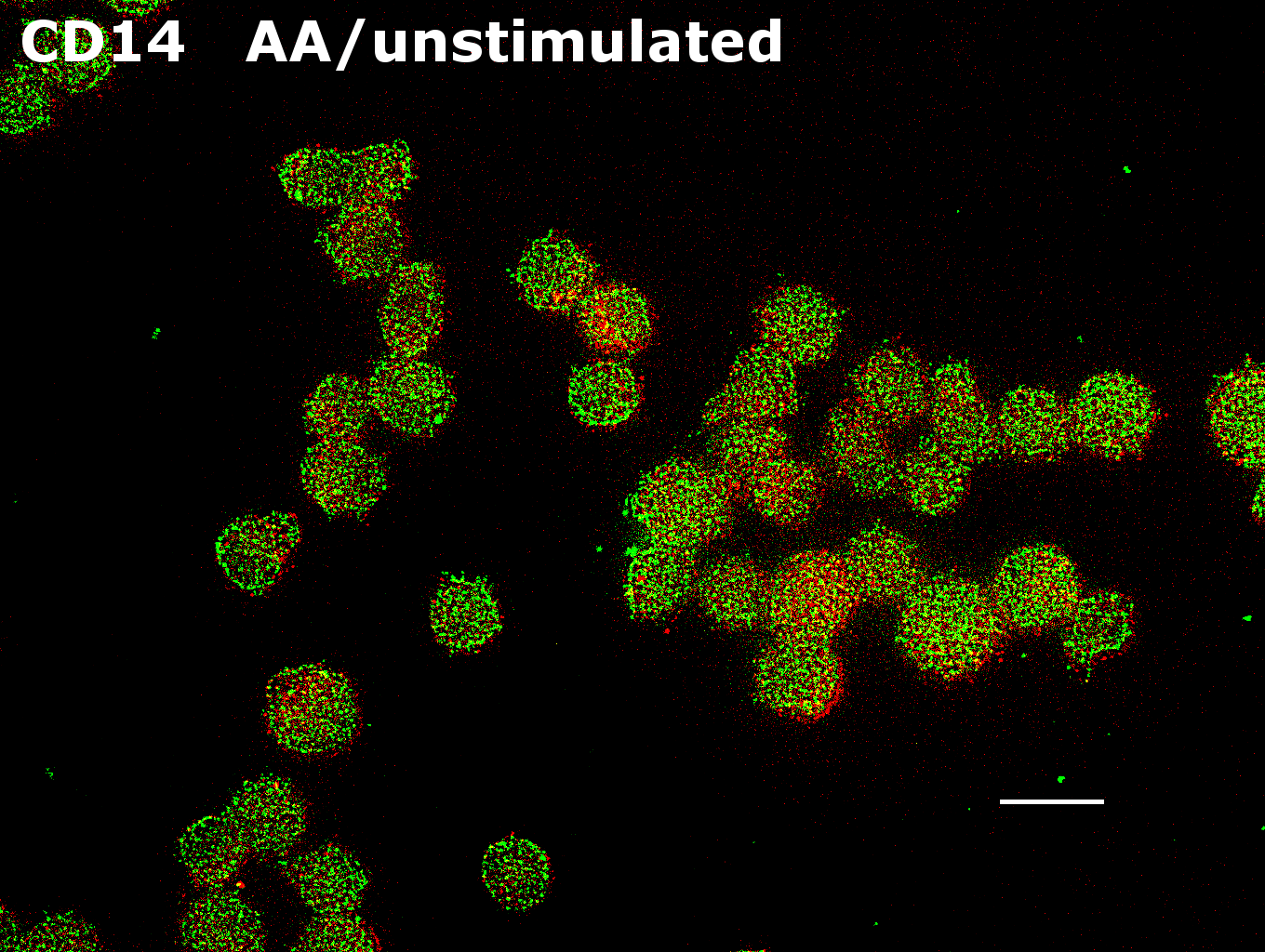

Supplement: Figure S19 — GM1 is labeled in red; CD14 is labeled in green. Scale bar represents 20 µm. Related to Fig. 2G. [file peerj-04-1663-s019.png]

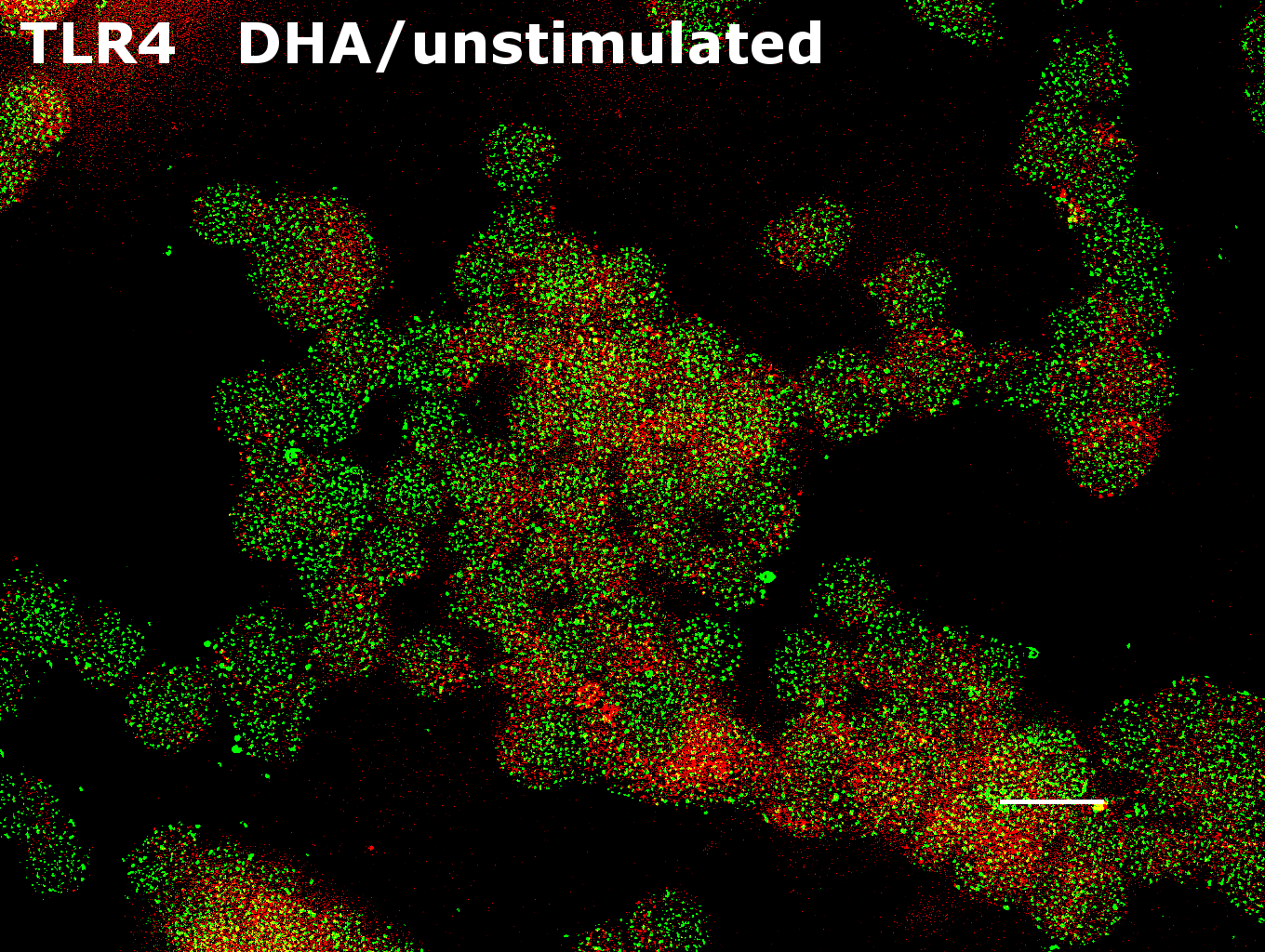

Supplement: Figure S20 — GM1 is labeled in red; TLR4 is labeled in green. Scale bar represents 20 µm. Related to Fig. 3A. [file peerj-04-1663-s020.png]

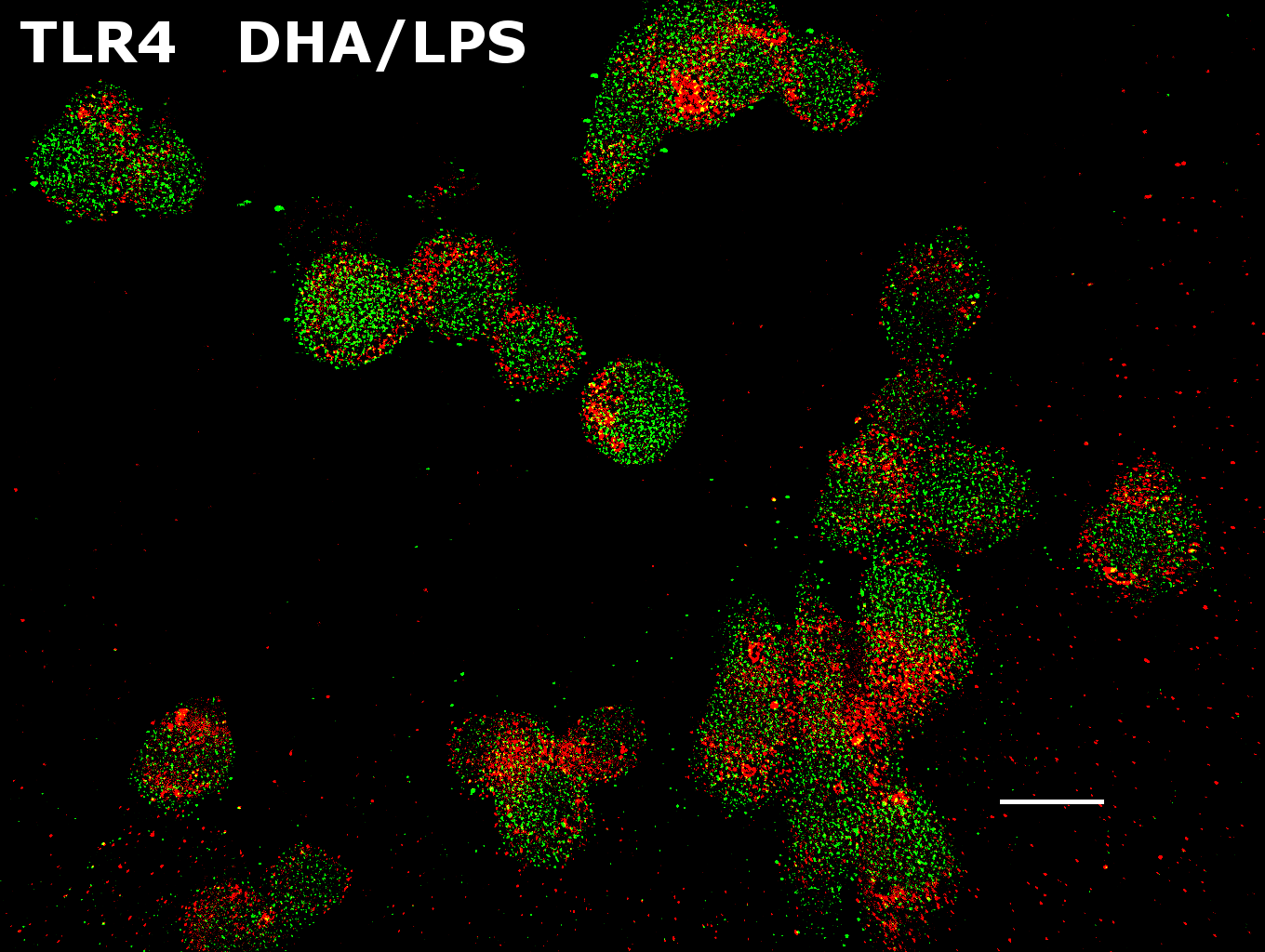

Supplement: Figure S21 — GM1 is labeled in red; TLR4 is labeled in green. Scale bar represents 20 µm. Related to Fig. 3A. [file peerj-04-1663-s021.png]

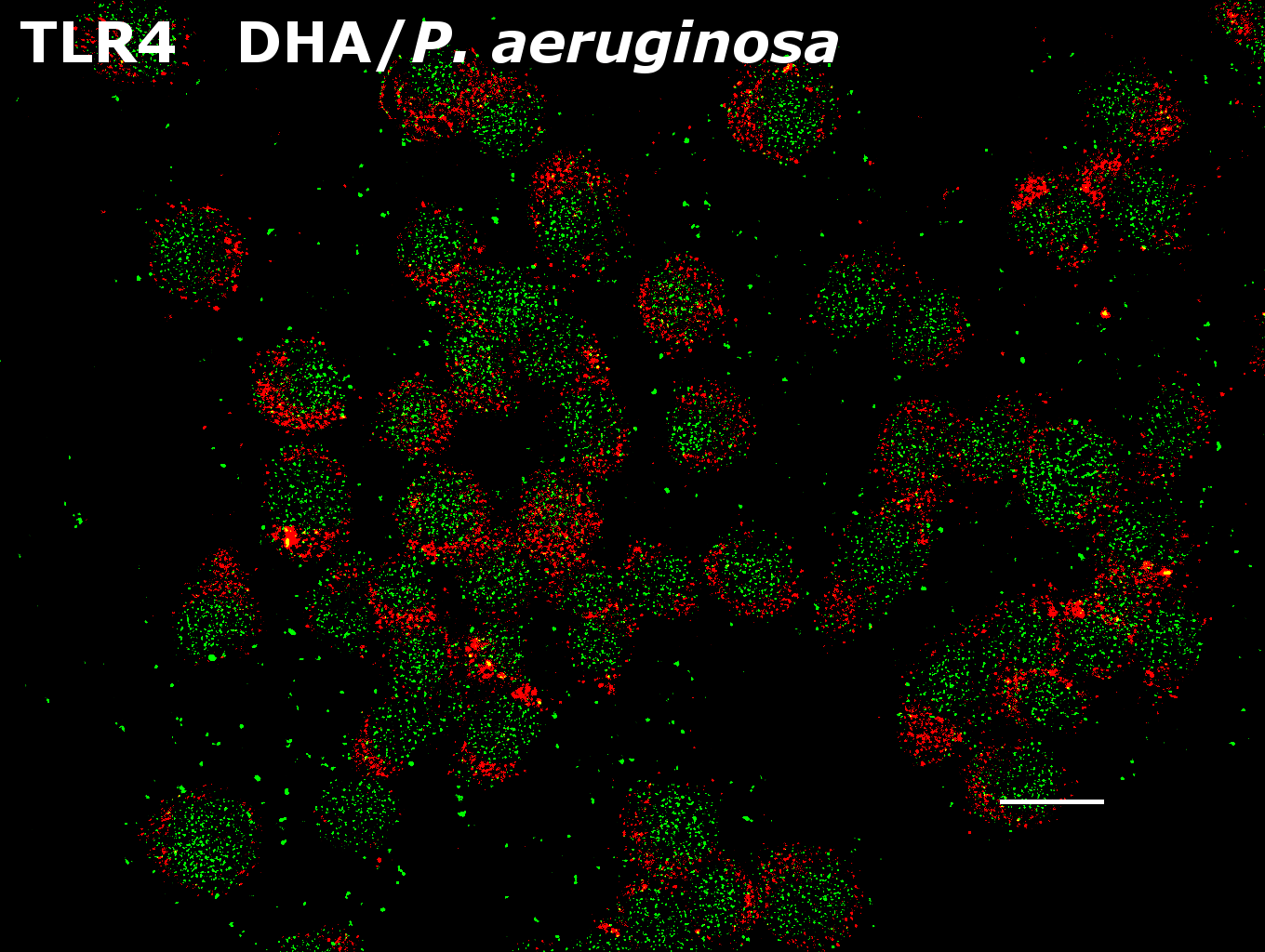

Supplement: Figure S22 — GM1 is labeled in red; TLR4 is labeled in green. Scale bar represents 20 µm. Related to Fig. 3A. [file peerj-04-1663-s022.png]

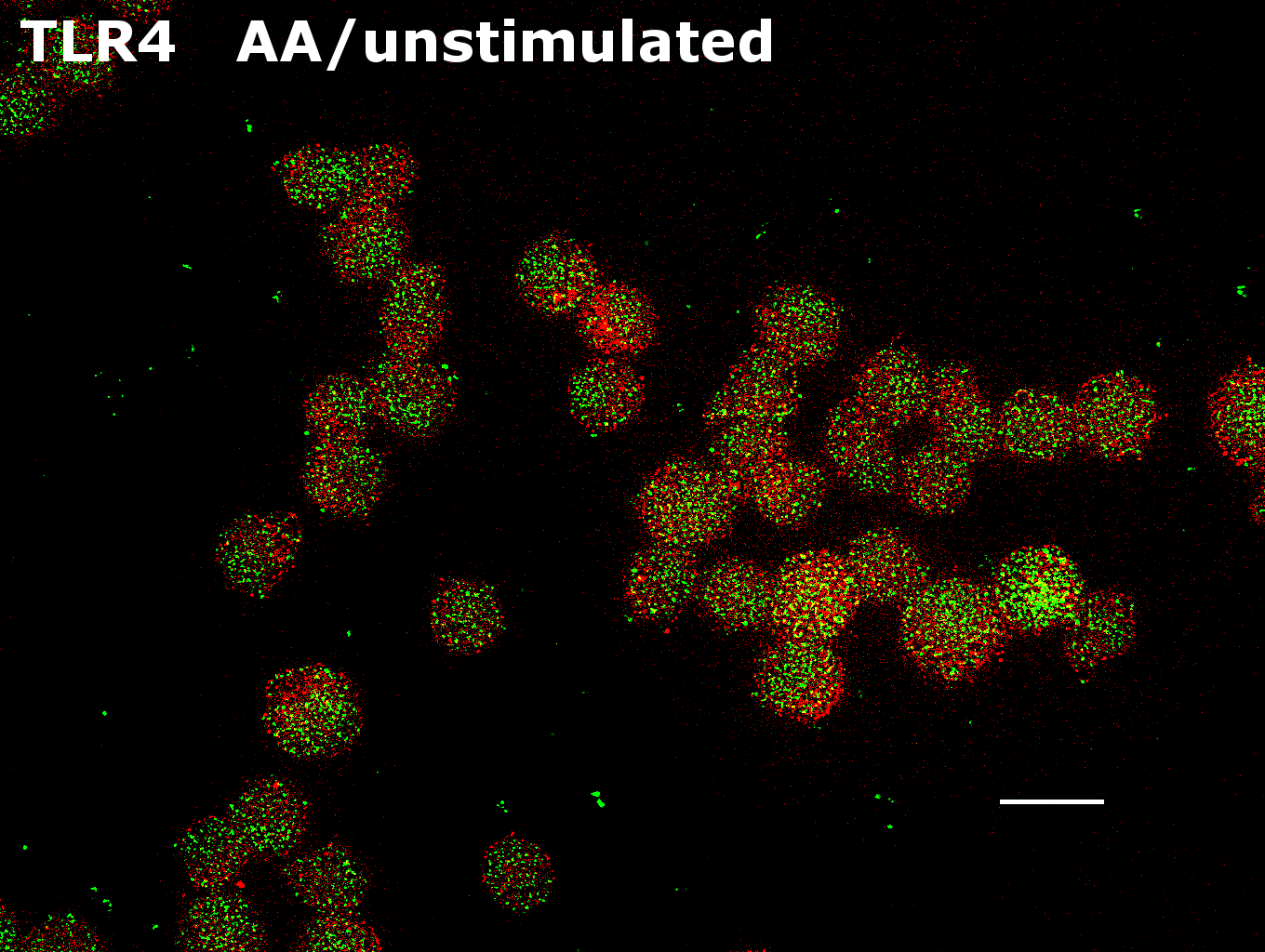

Supplement: Figure S23 — GM1 is labeled in red; TLR4 is labeled in green. Scale bar represents 20 µm. Related to Fig. 3A. [file peerj-04-1663-s023.png]

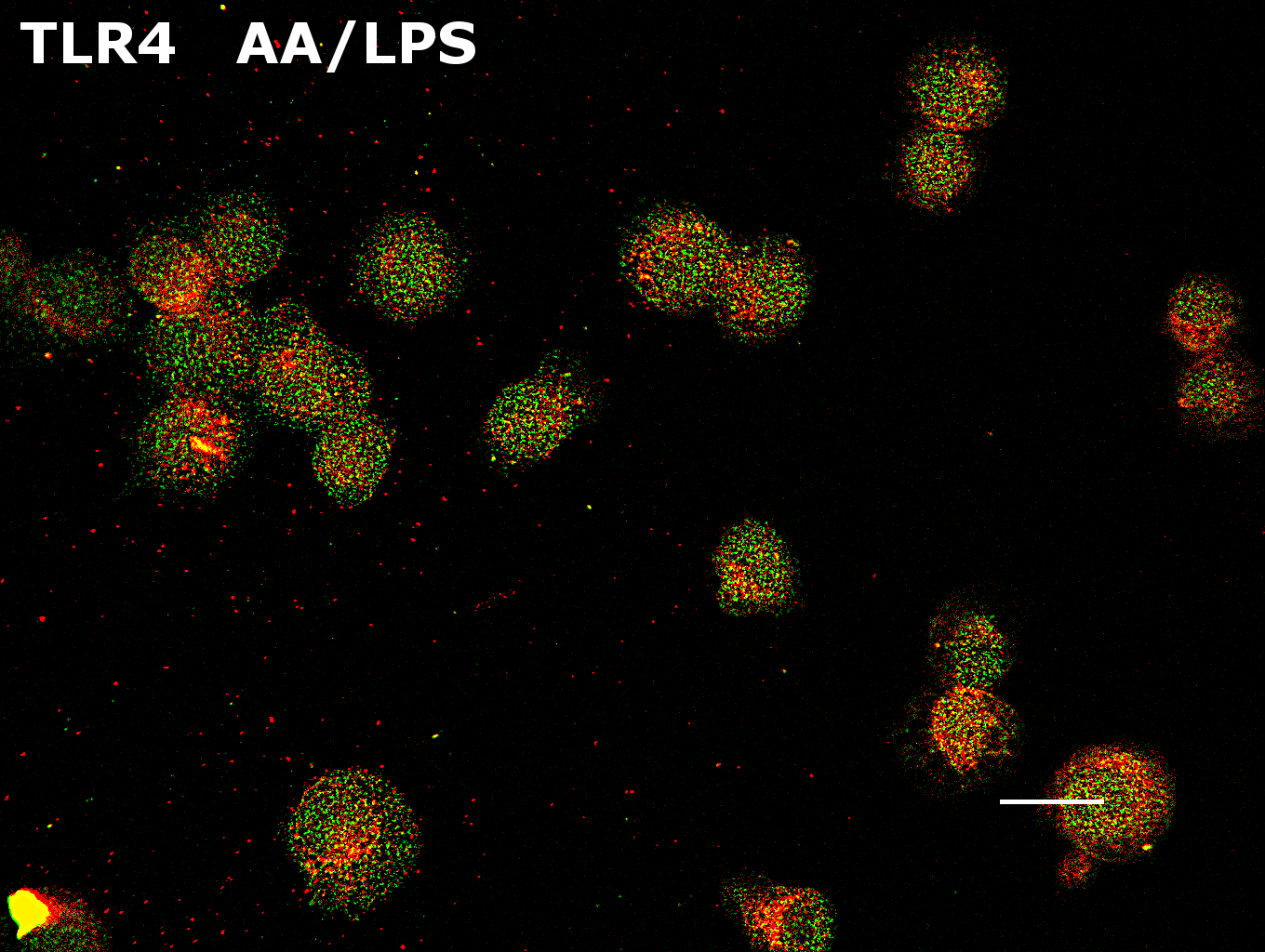

Supplement: Figure S24 — GM1 is labeled in red; TLR4 is labeled in green. Scale bar represents 20 µm. Related to Fig. 3A. [file peerj-04-1663-s024.png]

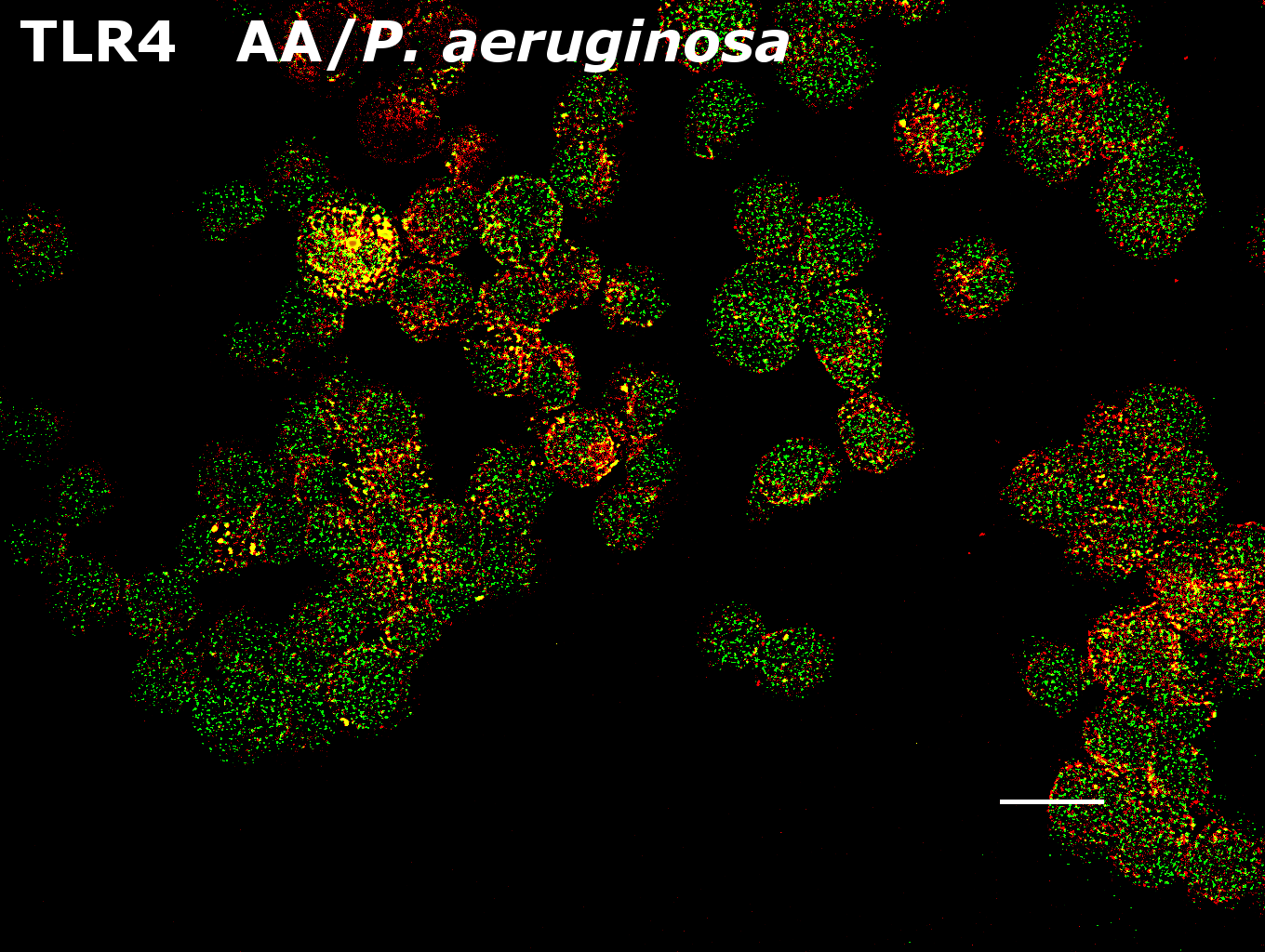

Supplement: Figure S25 — GM1 is labeled in red; TLR4 is labeled in green. Scale bar represents 20 µm. Related to Fig. 3A. [file peerj-04-1663-s025.png]

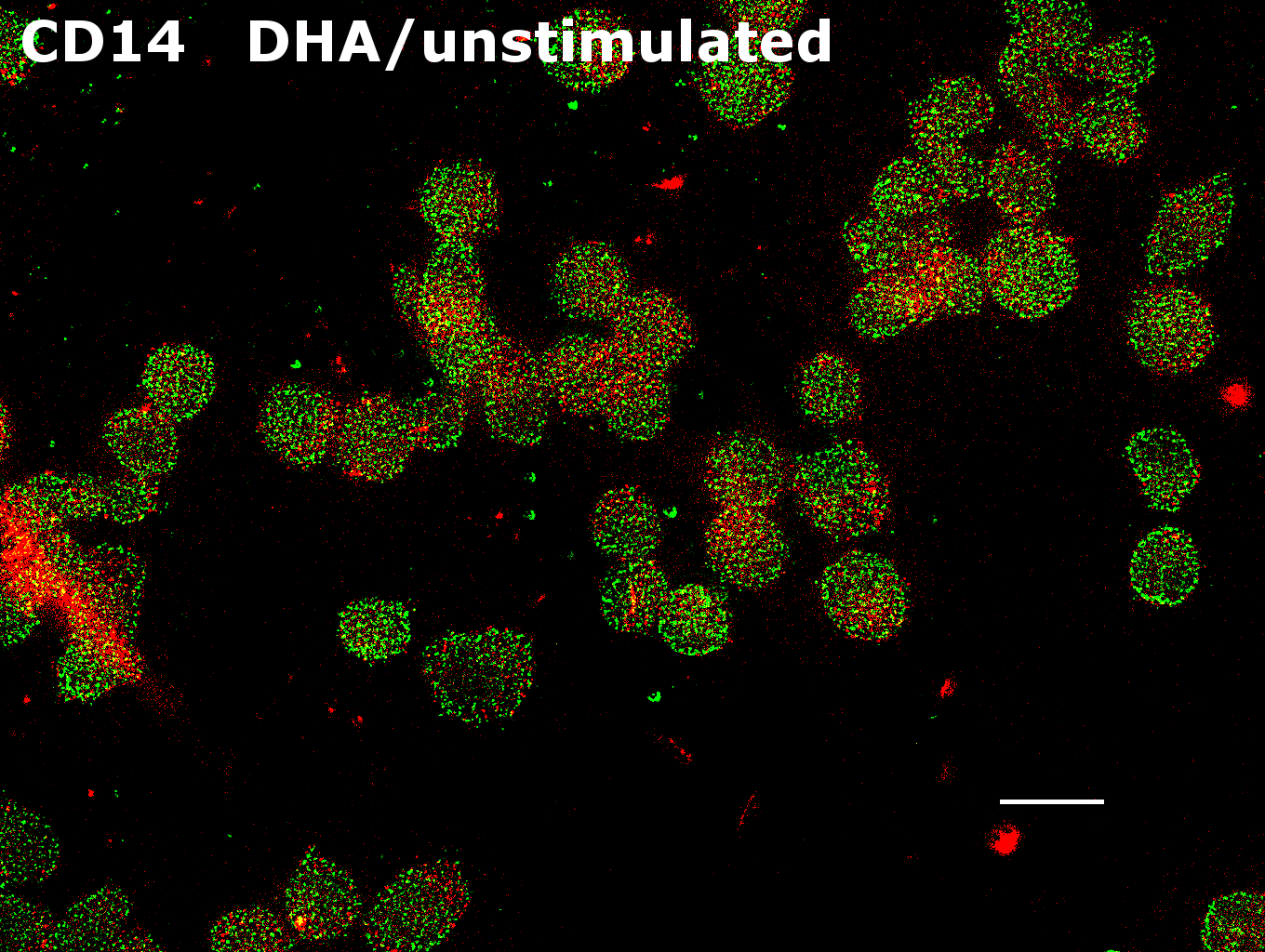

Supplement: Figure S26 — GM1 is labeled in red; CD14 is labeled in green. Scale bar represents 20 µm. Related to Fig. 3C. [file peerj-04-1663-s026.png]

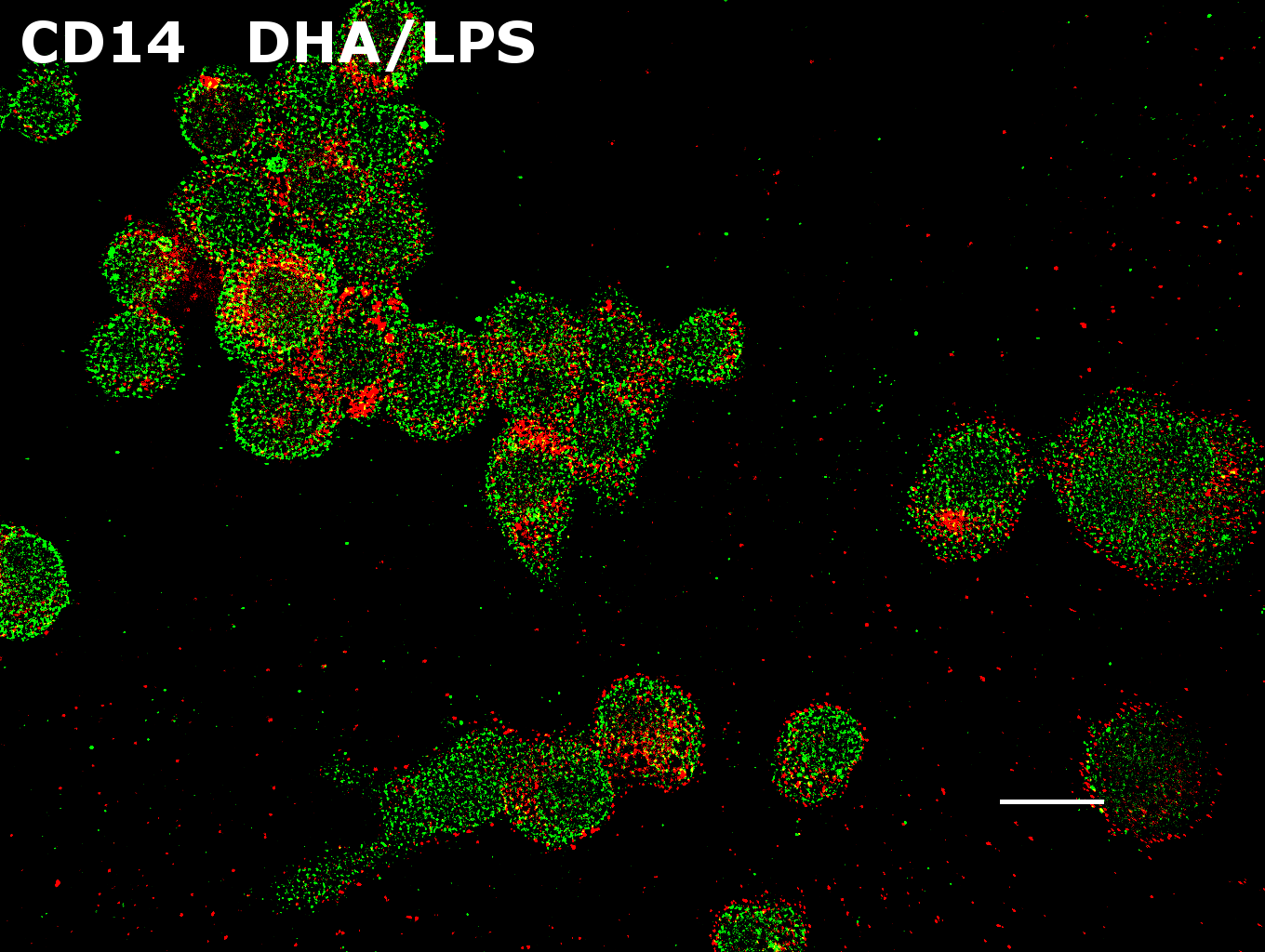

Supplement: Figure S27 — GM1 is labeled in red; CD14 is labeled in green. Scale bar represents 20 µm. Related to Fig. 3C. [file peerj-04-1663-s027.png]

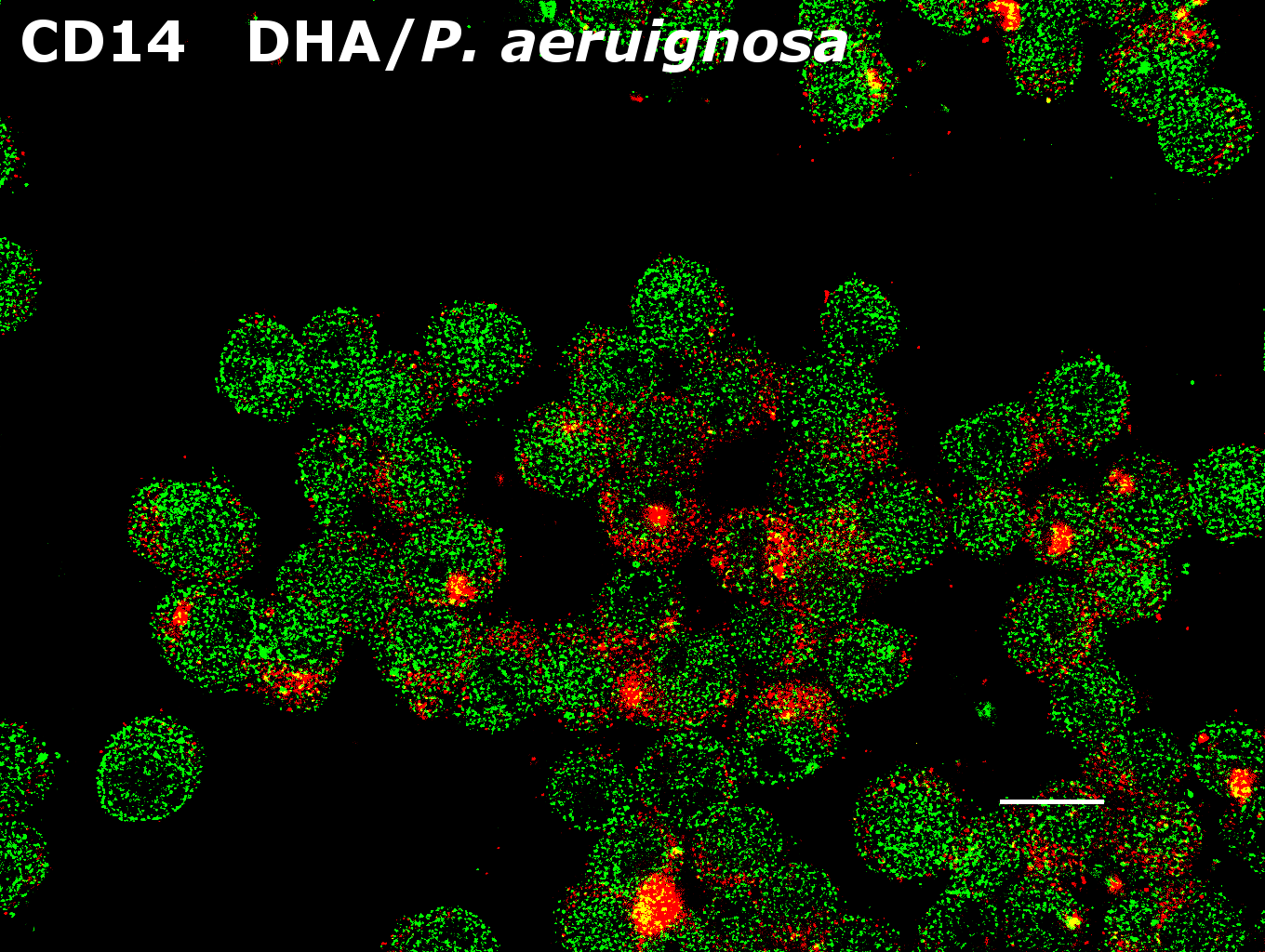

Supplement: Figure S28 — GM1 is labeled in red; CD14 is labeled in green. Scale bar represents 20 µm. Related to Fig. 3C. [file peerj-04-1663-s028.png]

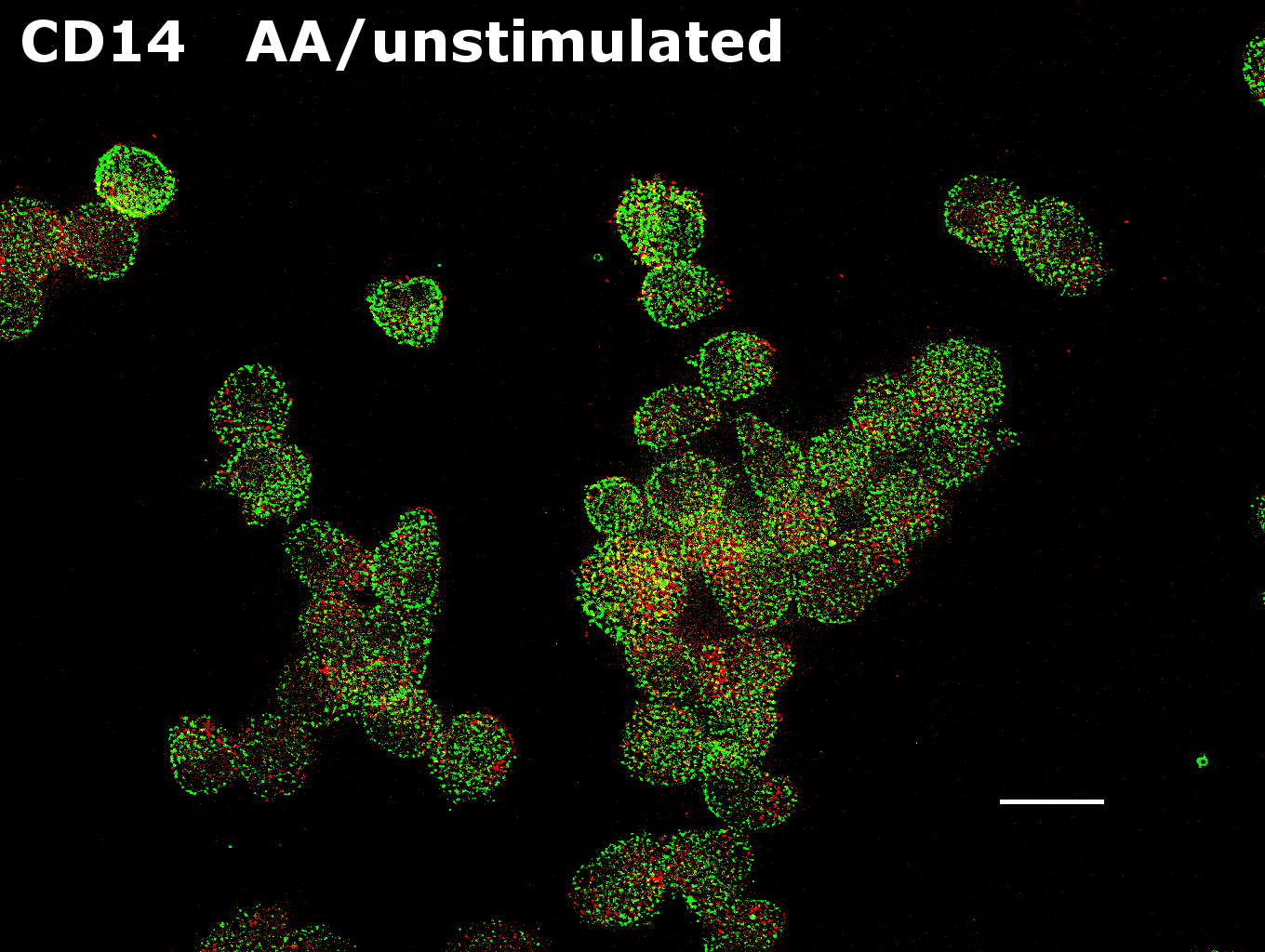

Supplement: Figure S29 — GM1 is labeled in red; CD14 is labeled in green. Scale bar represents 20 µm. Related to Fig. 3C. [file peerj-04-1663-s029.png]

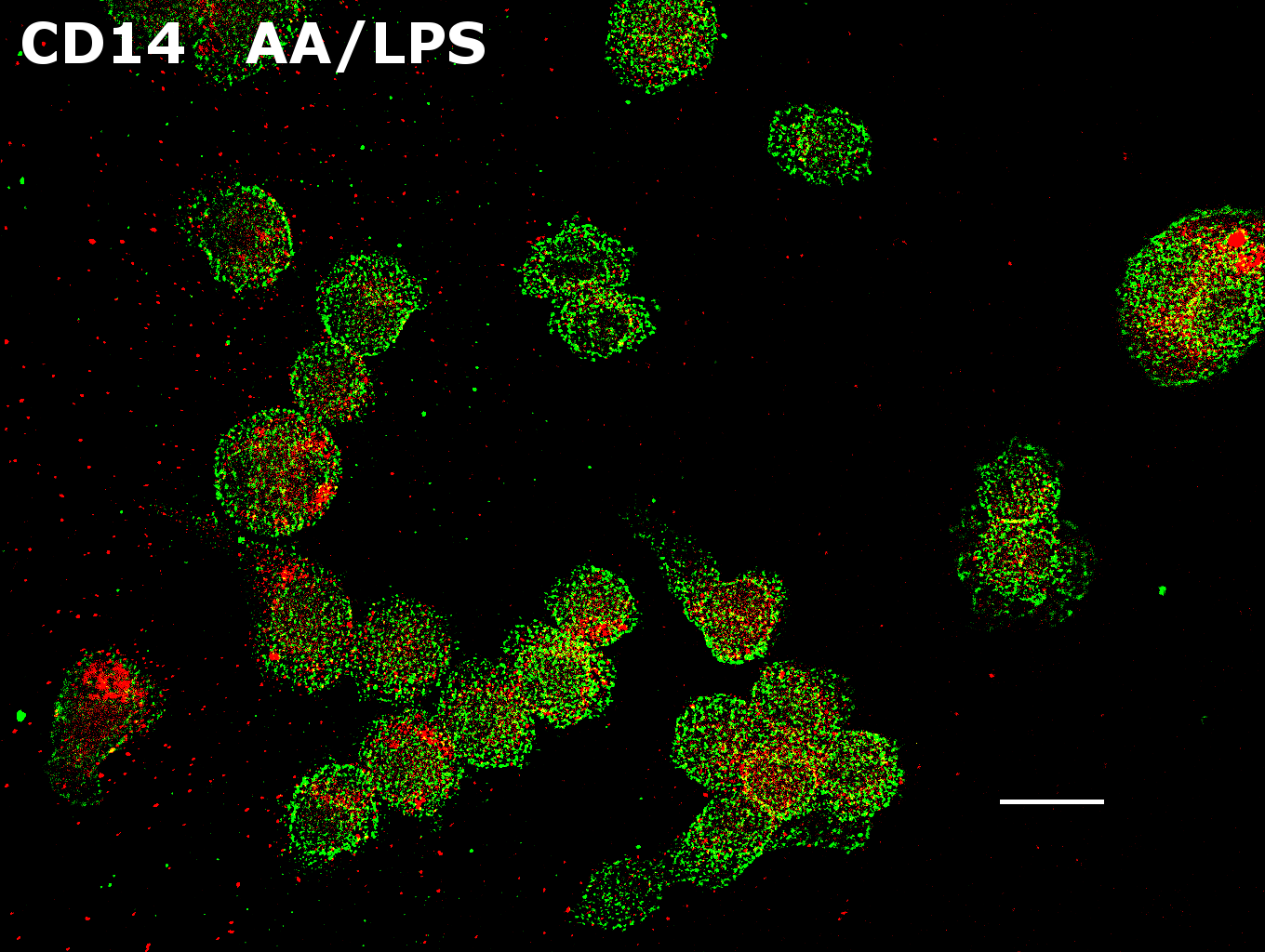

Supplement: Figure S30 — GM1 is labeled in red; CD14 is labeled in green. Scale bar represents 20 µm. Related to Fig. 3C. [file peerj-04-1663-s030.png]

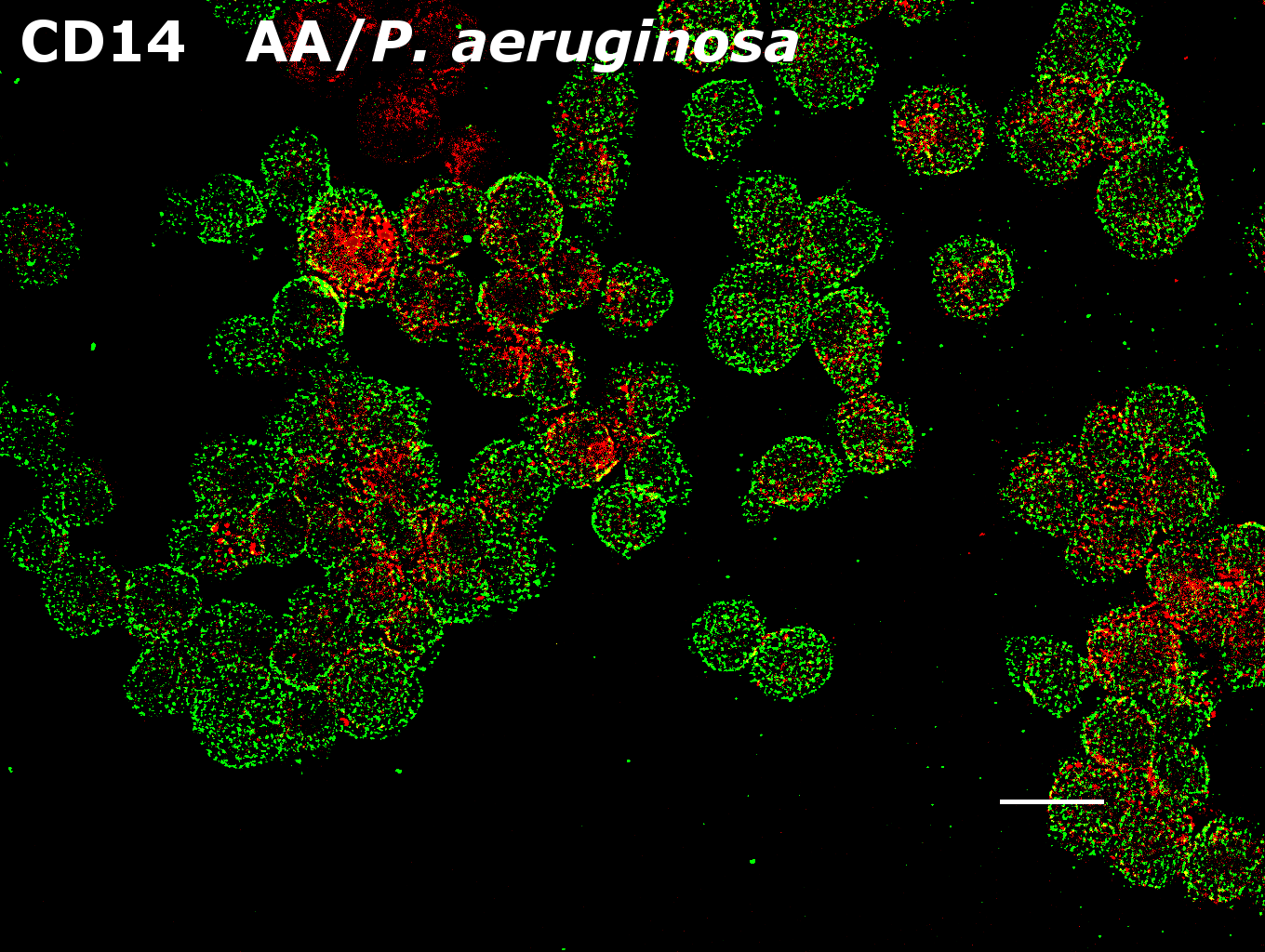

Supplement: Figure S31 — GM1 is labeled in red; CD14 is labeled in green. Scale bar represents 20 µm. Related to Fig. 3C. [file peerj-04-1663-s031.png]
